# Open-label pilot for treatment targeting gut dysbiosis in myalgic encephalomyelitis/chronic fatigue syndrome: neuropsychological symptoms and sex comparisons

**DOI:** 10.1186/s12967-018-1392-z

**Published:** 2018-02-06

**Authors:** Amy Wallis, Michelle Ball, Henry Butt, Donald P. Lewis, Sandra McKechnie, Phillip Paull, Amber Jaa-Kwee, Dorothy Bruck

**Affiliations:** 1 Psychology Department, College of Health and Biomedicine, Victoria University, Melbourne, Australia; 2 Bioscreen (Aust) Pty Ltd., Melbourne, Australia; 3 CFS Discovery Clinic, Donvale, Melbourne, Australia; 4 College of Engineering and Science, Victoria University, Melbourne, Australia

**Keywords:** Antibiotic, Chronic fatigue syndrome, Clinical outcomes, Gut dysbiosis, Microbiota-gut-brain, Myalgic encephalomyelitis, Neuropsychological symptoms, Open-label pilot, Probiotic, Sex comparisons, *Streptococcus*, Treatment

## Abstract

**Background:**

Preliminary evidence suggests that the enteric microbiota may play a role in the expression of neurological symptoms in myalgic encephalomyelitis/chronic fatigue syndrome (ME/CFS). Overlapping symptoms with the acute presentation of d-lactic acidosis has prompted the use of antibiotic treatment to target the overgrowth of species within the *Streptococcus* genus found in commensal enteric microbiota as a possible treatment for neurological symptoms in ME/CFS.

**Methods:**

An open-label, repeated measures design was used to examine treatment efficacy and enable sex comparisons. Participants included 44 adult ME/CFS patients (27 females) from one specialist medical clinic with *Streptococcus* viable counts above 3.00 × 10^5^ cfu/g (wet weight of faeces) and with a count greater than 5% of the total count of aerobic microorganisms. The 4-week treatment protocol included alternate weeks of Erythromycin (400 mg of erythromycin as ethyl succinate salt) twice daily and probiotic (d-lactate free multistrain probiotic, 5 × 10^10^ cfu twice daily). 2 × 2 repeated measures ANOVAs were used to assess sex-time interactions and effects across pre- and post-intervention for microbial, lactate and clinical outcomes. Ancillary non-parametric correlations were conducted to examine interactions between change in microbiota and clinical outcomes.

**Results:**

Large treatment effects were observed for the intention-to-treat sample with a reduction in *Streptococcus* viable count and improvement on several clinical outcomes including total symptoms, some sleep (less awakenings, greater efficiency and quality) and cognitive symptoms (attention, processing speed, cognitive flexibility, story memory and verbal fluency). Mood, fatigue and urine d:l lactate ratio remained similar across time. Ancillary results infer that shifts in microbiota were associated with more of the variance in clinical changes for males compared with females.

**Conclusions:**

Results support the notion that specific microorganisms interact with some ME/CFS symptoms and offer promise for the therapeutic potential of targeting gut dysbiosis in this population. *Streptococcus* spp. are not the primary or sole producers of d-lactate. Further investigation of lactate concentrations are needed to elucidate any role of d-lactate in this population. Concurrent microbial shifts that may be associated with clinical improvement (i.e., increased *Bacteroides* and *Bifidobacterium* or decreased *Clostridium* in males) invite enquiry into alternative strategies for individualised treatment.

*Trial Registration* Australian and New Zealand Clinical Trial Registry (ACTRN12614001077651) 9th October 2014. https://www.anzctr.org.au/Trial/Registration/TrialReview.aspx?id=366933&isReview=true

**Electronic supplementary material:**

The online version of this article (10.1186/s12967-018-1392-z) contains supplementary material, which is available to authorized users.

## Background

### Background and objectives

ME/CFS is a complex, neuroimmune condition characterised by post-exertional mental and physical fatigue that is disproportionate to the level of exertion [1]. The multisystemic dysregulation results in pathophysiological abnormalities affecting a combination of central nervous, immune, gastrointestinal, energy metabolism, cardiovascular and respiratory systems manifesting in heterogeneous symptomatic presentations [1]. The history of diagnostic discrepancies (see [1–4]) is reflected in varied prevalence rates between .08 and 2.6% [5–11] but the burden on both the patient, their family and society is unequivocal [12]. This burden is not only a result of the devastating impact that the condition has on the patient’s daily, occupational and social functioning [13–15] but can also be attributed to the direct cost of medical care that is often exacerbated by misdiagnosis and unclear treatment pathways [16, 17]. This awareness provides the rationale to examine the efficacy of treatments targeting pathophysiological abnormalities in ME/CFS patients with the hope of minimizing clinical exploration and identifying subgroups that may be more responsive to specific treatments.

Gastrointestinal disturbance and comorbid irritable bowel syndrome (IBS) are common in ME/CFS [18]. Estimates from a clinical sample of 1400 patients found that 80–90% experienced recurring gastrointestinal symptoms [19]. Intestinal permeability of the mucosal lining of the gastrointestinal tract [20, 21] and an imbalance in commensal enteric bacteria (i.e., gut dysbiosis) using culture-based methods (i.e., microbiota [22, 23]) and DNA sequencing (i.e., microbiome [24–26]) have also been shown in this population. These imbalances in both the microbiota and microbiome appear distinct from healthy controls [24, 26], and associated with inflammation [25] and symptom expression [23, 26–29]. Accumulating evidence suggests that microbial imbalances (whether consequential or causative) should not be viewed in isolation as they may be relevant for multiple ME/CFS symptoms, including but not limited to neurological manifestations.

Gut–brain interaction occurs through multiple bidirectional pathways including through central, autonomic, and enteric nervous systems; neuroendocrine and neuroimmune pathways; and enteric microbiota [30–32]. Our understanding of the importance of the symbiotic relationship between enteric microbiota and health is becoming well accepted, with research efforts directed towards understanding mechanisms of microbial/host communication (see [33]). Gut dysbiosis may directly or indirectly precede gastrointestinal, neurocognitive and immune disturbances [34] or may be a consequence of stress and neurobiological mechanisms (e.g., in animal models [35–37]). Results of antibiotic [27], probiotic [38–40] and faecal transplant [41] interventions provide preliminary support for microbiota–gut–brain interactions in ME/CFS.

The d-lactate theory has been proposed as a possible mechanism for the neurological disturbances associated with gut dysbiosis in this population [23, 34, 42]. d-Lactic acidosis is an acute metabolic acidosis with associated encephalopathy that is observed in patients with a history of small bowel resections [43]. The shortened small bowel can lead to impaired absorption of carbohydrates, preferential growth of selected gut bacteria (e.g., increase in some species of *Lactobacillus* and *Streptococcus*) that promotes an acidic colonic environment and excess production of d-lactic acid [44]. This abundance of d-lactic acid combined with decreased metabolic capacity can lead to excess absorption within the blood and brain believed to play a role in the neurological symptoms of d-lactic acidosis [44]. Within ME/CFS, an overgrowth of *Streptococcus* and *Enterococcus* species (d-lactic acid producing bacteria) has been observed in culture-based microbial studies [23]. This bacterial imbalance, combined with overlapping neurological symptoms and possible mechanisms have contributed to the proposal that subclinical concentrations of d-lactate may play a role in ME/CFS presentations [42]. To date, measurement of d-lactate concentrations in ME/CFS have not been published.

In accordance with the d-lactate theory, an antibiotic treatment has been proposed to target the overgrowth of commensal enteric microbiota within the *Streptococcus* genus. Results from our group’s earlier pilot showed initial promise on some sleep and mood outcomes for a subgroup of participants who decreased in *Streptococcus* after 6 days of oral erythromycin treatment [27]. Other probiotic interventions used with ME/CFS patients may contradict the d-lactate hypothesis. Results indicating improved neurocognitive [38] and anxiety [39] symptoms using lactic acid-producing bacteria (predominantly *Lactobacillus* strains) question the mechanisms at play. Notably, colonic bacteria can produce d- and l-lactate with the ratio and rate of metabolism dependent on the species [45]. The proportion of d:l lactate produced by the bacterial strains used in the probiotic studies were not measured. The validity of the d-lactate theory as well as the efficacy of antibiotic and probiotic interventions in ME/CFS requires further examination.

Findings from our cross-sectional study correlating commensal microbiota and clinical symptoms in 274 ME/CFS patients [28] provided an interesting perspective on the role of d-lactate in males and females. Results showed small to moderate positive correlations for both *Streptococcus* and *Lactobacillus* with symptoms in males, suggesting that increased abundance of these genera were related to more impairment across several ME/CFS symptoms [28]. For *Streptococcus,* opposite associations were shown in females with small negative correlations suggesting that higher *Streptococcus* was associated with less pain, neurosensory and immune symptoms. These results highlighted the importance of considering sex differences in microbial function and supported the notion of the ‘microgenderome’, i.e. the critical role of sex hormones on host–microbiota interactions [46]. These findings also raised questions about the possibility of sex differences in response to oral erythromycin treatment targeting an overgrowth of *Streptococcus*.

This study is positioned within the context of d-lactate and microgenderome theories. In light of research yielding sex-specific correlations between *Streptococci* and ME/CFS symptoms [28], the objective of this study was to examine whether there was a sex-specific treatment response to an intervention designed to reduce the content of bacteria of the *Streptococcus* genus. Thus this pilot study compares the treatment response of male and female ME/CFS patients using a combined antibiotic and probiotic intervention aimed at reducing *Streptococcus.* Clinical outcomes measuring sleep, mood and cognitive symptoms were prioritised. The intervention was an extension of the earlier pilot [27] with alternate weeks of oral erythromycin and d-lactate-free probiotic supplementation across a 4-week period. Urine d-lactate and l-lactate concentrations were also measured to observe variation of lactate levels with this intervention to understand possible mechanisms of microbial–gut–brain interactions. To enable sufficient sample sizes for sex comparisons, an open-label design was used with the primary feasibility objective of determining the appropriateness of the intervention for both sexes rather than placebo control.

## Methods

### Trial design and participant recruitment

This open-label, non-randomised pilot used a repeated measures design with a baseline, intervention and post-intervention protocol across 6 weeks (see Table 1). The prospective intention was to recruit 40 patients with equal proportions of males and females to enable sex comparisons. Screening and recruitment was continuous, with consecutive commencement dates according to patient presentation at CFS Discovery Clinic, Melbourne, Australia.

**Table 1:** Trial design

Week		1	2	3	4	5	6
Day		1–7	8–14	15–21	22–28	29–35	36–42

Phase	Screening/recruitment	Baseline	Intervention				Post-intervention
Treatment prescribed			Antibiotic Erythromycin 400 mg/b.d.	Probiotic Pro4-50 d-Lactate Free Multistrain 1 tablet b.d.	Antibiotic Erythromycin 400 mg/b.d.	Probiotic Pro4-50 d-Lactate Free Multistrain 1 tablet b.d.	
Measures/ monitoring	• Consent forms • Screening and background questions • Stool and urine collection	*Day 1* • Cognitive test battery *Days 1*–*7* • Actigraphy • Sleep diary • MAC • MFTQ *Day 7* ^a^ • SSH • DASS-21 • MFI-20 • POMS • PSQI • ISI	*Day 14* • Phone call, symptom monitoring	*Day 21* • Phone call, symptom monitoring	*Day 28* • Phone call, symptom monitoring	*Day 35* • Phone call, symptom monitoring	*Day 1* • Cognitive test battery *Days 1*–*7* • Actigraphy • Sleep diary • MAC • MFTQ • Stool and urine collection *Day 7* ^a^ • SSH • DASS-21 • MFI-20 • POMS • PSQI • ISI

^a^Participants had the option of completing these scales at any time during Baseline and Post-intervention phases if they wanted to reduce the risk of post-exertional mental fatigue

New or current patients at the clinic aged above 18 years who met Canadian Consensus diagnostic Criteria for ME/CFS [47] were invited to be screened for participation in this study. Eligible participants were patients with *Streptococcus* viable counts above 3.00 × 10^5^ cfu/gm and more than 5% of the total count of aerobic microorganisms. Participants were asked to refrain from taking other antibiotics (from 4 weeks prior), probiotics (from 2 weeks prior), and substantially altering their diet, prescription medications or over-the-counter supplements across the screening and trial period. Known adverse reactions, contra-indications to the treatment protocol and/or significant comorbid physical or psychiatric illnesses excluded participation.

Trial methods were conducted in accordance with the guidelines for human experimental research and the Australian Clinical Trial Handbook [48]. Ethics approval was obtained from Victoria University Human Research Ethics Committee in June 2015 (HRE15-010). Additional trial details are available on the Australian and New Zealand Clinical Trial Registry (ACTRN12614001077651).

### Intervention

The treatment protocol combined antibiotic and probiotic therapy taken on alternate weeks. Tablets of Erythromycin 400 mg were given twice daily during weeks 2 and 4 (Erythromycin was given as the Ethyl Succinate salt and supplied by Amdipharm Mercury Pty Ltd or by Alphapharm Pty Ltd). Two capsules of Pro4-50 d-lactate free multistrain probiotic (Spectrumceuticals Pty Ltd, Belrose, New South Wales, Australia) were taken daily during weeks 3 and 5. Each probiotic capsule contained *Lactobacillus rhamnosus* (2.5 × 10^10^ cfu), *Bifidobacterium lactis* (1.5 × 10^10^ cfu), *Bifidobacterium breve* (5 × 10^6^ cfu), *Bifidobacterium longum* (5 × 10^6^ cfu). The off-label use of Erythromycin required notification to the Therapeutic Goods Administration under the Clinical Trial Notification scheme (Trial Number: 2015/0492) and approval was obtained on 29 June 2015.

Participants completed the intervention in their own homes. Compliance and adverse events were monitored with weekly phone calls throughout the intervention phase and participant completion of treatment adherence schedules.

### Outcomes

Table 1 provides an overview of the timing of the outcomes assessed. Sleep patterns were measured objectively (actigraphy) using wrist Actiwatch monitors (Respironics Actiwear 2) that estimate movement and light. Participants completed a *Response Booklet* that included the sleep diary and self-report scales. Participants attended two external appointments for administration of the Cognitive Test Battery. The Cognitive Test Battery included measures of attention, memory, verbal fluency and executive functioning (see Additional file 1: Additional Method for additional details of all clinical measures and selected outcome variables).

The faecal microbial counts were performed on specimens that were preserved by cooling and then controlling the temperature until the commencement of laboratory analysis (see Additional file 1: Additional method). Classical cultural methods, on a variety of media, were used to perform the counts (see [28] for details of microbial identification and microbial quantification procedures). Identification of bacteria was performed by Matrix Assisted Laser Absorption and Ionisation Time of Flight Mass Spectrometry (MALDI-TOF–MS) using a proprietary peptide data base (MALDI Biotyper Bruker Daltonics, Bremen, Germany). Microbial variables included the count and relative abundance (RA) of selected aerobic (*Streptococcus*, *Enterococcus*, *Escherichia*) and anaerobic bacteria (*Bacteroides*, *Bifidobacterium*, *Clostridium*, *Eubacterium*, *Lactobacillus*). These variables were selected based on prior research [28]. RA_total_ was calculated by the ratio of each genus count divided by total detectable bacteria count (aerobic and anaerobic). The proportion of *Streptococcus* within total aerobic bacteria count (RA_aerobe_) was also used as an outcome measure to be consistent with inclusion criteria and aid clinical interpretation.

The d-lactate and l-lactate concentrations in the urine samples were determined using High Performance Liquid Chromatography and Triple Quadrupole Mass Spectrometry (HPLC-TMS). Briefly, urine samples were acidified with hydrochloric acid and extracted with ethyl acetate. The ethyl acetate extracts were evaporated in a centrifugal vacuum evaporator. The residues were derivitised with an optically active reagent, (+)-O,O-diacetyl-l-tartaric anhydride, as originally described by Scheijen et al. [49]. These data are presented as the ratio of the concentrations of d-lactate to l-lactate. It is common to determine the ratio of analyte concentration in the urine sample the concentration of creatinine in the sample in order to correct for dilute or concentrated urine samples that arise from variation in the state of hydration of the subject. This was considered inappropriate in the current trial because there is evidence that the excretion of creatinine is increased in subjects suffering from ME/CFS (see [50]).

### Primary and secondary outcomes

Primary and secondary endpoints were the change in scores on psychological outcomes at post-intervention for the intention-to-treat (ITT) population (i.e., all participants who commenced at baseline). A priori allocation of primary outcome status was based on evidence from research indicating sensitivity measuring treatment effects in this [27] and other clinical populations [51]. Primary outcome variables included a measure of sleep (actigraphic sleep efficiency; SE), mood (Profile of Mood States-Short Form Total Mood Disturbance, POMS [52]) and a measure of sustained visual attention (Rapid Visual Processing-A′, RVP-A′ from the Cambridge Neuropsychological Test Automated Battery, CANTAB [53]).

Multiple secondary endpoints were selected to evaluate change in microbiota (*Streptococcus*, *Bifidobacteria* and *Lactobacillus* count and RA), urinary d-lactate (d:l lactate ratio) and clinical symptoms including: objective sleep symptoms [Actigraphy sleep onset latency (SOL), wake after sleep onset (WASO), and restlessness/sleep fragmentation index (SFI)]; subjective sleep symptoms (Sleep Diary SOL, WASO, SE, and the Pittsburgh Sleep Quality Index, PSQI—Global Score [54]); mood (Depression, Anxiety and Stress Scale, DASS-21 [55]); cognition (word memory, story memory, spatial working memory, visual learning, verbal fluency, processing speed, cognitive flexibility and planning); fatigue (General Fatigue subscale from the Multidimensional Fatigue Inventory, MFI-20 [56]); and the *Brain Fog* subscale of the Multiple Fatigue Types Questionnaire, MTFQ [57]); and total symptoms (Symptom Severity and Symptom Hierarchy Profile, SSH-Total score [47]).

Uncertainty about the suitability of endpoints suggested a less hierarchical approach to outcome classification. Subsequently, the results of both primary and secondary outcome variables are presented together prioritising outcomes with large effect sizes (ES).

### Sample size

The study aimed to recruit equal proportions of males and females to conduct sex comparisons. Power analyses conducted by G*Power 3.1 indicated that the minimum sample size of n = 20 per group (alpha = .05, power = .8) would enable moderate to large ES estimates to achieve significance using analysis of variance (2 × 2 repeated measures ANOVA). A sample size of 40 for the combined group (alpha = .05, power = .8) was required to identify significant, moderate ES estimates using repeated measures ANOVA within factors (f = .23).

### Statistical methods

#### Group comparisons for primary and secondary outcomes

Using SPSS version 22.0 [58], mixed between-within subjects analysis of variance (2 × 2 repeated measures ANOVA) assessed the sex-time interaction effect and main effects (time and sex) for each outcome. These were performed for the whole sample (according to ITT protocols). Cases with missing data were excluded for pairwise analyses to retain maximum representation for each variable.

##### Focus on effect estimates

As encouraged by the CONSORT guidelines, it was decided to prioritise estimates of ES values and their confidence intervals (CI) [59]. Partial eta squared $\left( {\mu_{p}^{2} } \right)$ values are reported as the ES estimate produced by ANOVA analyses in SPSS software. Cohen’s [60] guidelines for interpreting partial eta squared were employed (small = .01, moderate = .06, large = .14). A conservative approach was used to avoid over-interpretation and the risk of Type 1 errors with multiple outcomes. Therefore, only outcomes with large effect sizes were used to examine treatment efficacy. Wuensch’s [61] explanations and Smithson’s syntax scripts for use in SPSS software were used to obtain 90% ES confidence intervals. Wuensch [61] explains that 90% confidence intervals are preferred because they are consistent with the ANOVA results and the .05 criterion of statistical significance. Additionally, partial eta squared values can only be positive values and a 95% confidence interval can include negative values. Exact significance values (*P*) are provided without use of Bonferroni corrections or the dichotomous categorisation of significance levels.

### Assumptions: tests used and managing violations

#### Normality

Each outcome variable was assessed for normality using the Shapiro–Wilk test in SPSS. Mild violations in normality were seen across several clinical variables, microbial RA and d:l lactate ratio variables. These variables were not transformed in accordance with criticisms of using transformations in psychosocial and biomedical research [62].

However, large violations in normality were seen on all microbial count variables. The nature of exponentially large values provided the rationale to transform these variables. Log10 transformations were applied and resulted in improvements in normality. Results were back-transformed after analysis and presented in the original scale as recommended [63].

Parametric tests were performed with minor violations of normality after considering that (a) ANOVA is robust to violations of normality for samples larger than 30 [64] and (b) nonparametric alternatives (Wilcoxon Signed Rank and Sign Test) exclude ties and, therefore, oppose the theoretical premise of ITT analyses. Means and standard deviations at baseline and post are presented based on cases with pairwise comparisons in each 2 × 2 ANOVA (see Additional file 1: Table S1). In order to address possible concerns about the spread of scores and appropriately describe the data, median and range scores for ITT data at baseline and post are presented in Additional file 1: Table S2.

##### Homogeneity tests

Homogeneity tests were calculated during repeated measures ANOVA procedures. The Levene’s test was used to determine equality of error variances. Given that *p* values are provided, violations of this assumption (*p* < .05) are highlighted in Additional file 2: Table S3 to attempt to mitigate inaccurate interpretation. For readers focusing on probability statistics, it is recommended to use a more stringent interpretation of significance values for interaction and main effects when the Levene’s test is violated (i.e., *p* < .01; [64]). The Box’s *M* test was used to determine if the assumption of homogeneity of intercorrelations was met (*p* ≥ .001; [65]).

### Ancillary exploratory analyses: correlations

The results of primary analyses indicated the need for further investigation to understand outcomes and examine interactions between change in bacteria and change in symptoms. Correlations were chosen as the preferred method due to restrictions with sample size and violations of assumptions with other statistical techniques (i.e., MANOVA or regression). Proportional change scores were created for each clinical, microbial count and d:l lactate ratio variable using Eq. (1).

1 $$X_{Change} = \frac{PostX}{PreX} \times 100$$

where *X* represents each variable analysed.

Therefore, scores of 100 reflect no change at post and numbers above or below reflect an increase or decrease at post, respectively. Spearman’s rho correlations (*r*_s_) between change in clinical variables and change in microbial variables were chosen due to violations in normality. Missing cases were excluded pairwise. To allow for consistent interpretation of correlations, some *r*_s_ values were reversed (multiplied by − 1) so that a decrease in the clinical outcome score always represented improvement. Correlations were classified as small (.01), moderate (.03) and large (.05) effect sizes [66]. Only large effect sizes (i.e., *r*_*s*_ > .05) were interpreted to reduce the risk of Type 1 errors from multiple correlations.

## Results

### Participant recruitment and demographics

**Fig. 1:**
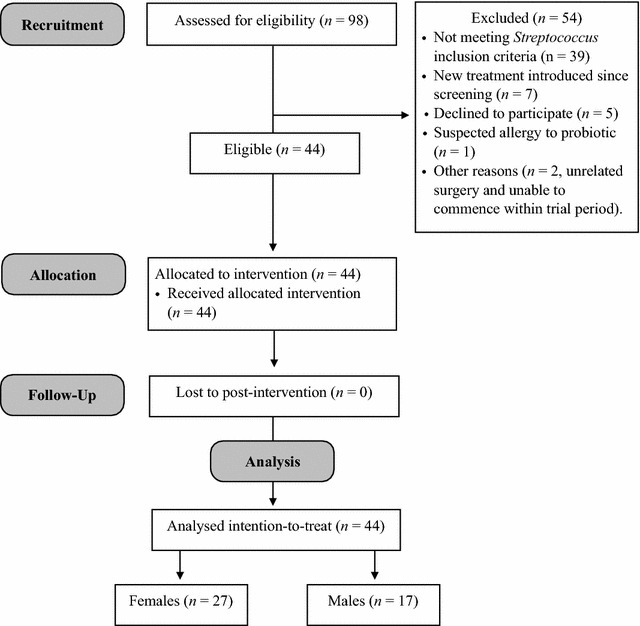
Participant flow diagram

Figure 1 shows the participant flow diagram with 44 patients deemed eligible and consenting to participate from the 98 screened during recruitment (44.9%). A predominance of females (*n* = 27) were recruited compared with males (*n* = 17). The recruitment period was between 29th July 2015 and 8th November 2016. The date of the last data collection was 26th December 2016. All participants completed both baseline and post-intervention stages.

Baseline demographics for all participants are presented in Table 2. Participants were aged between 18 and 65 years with mean ages similar between the sexes. On average females spent less time working per week with 15/23 females (65.2%) not working compared with 5/14 males (35.7%). Higher mean scores of baseline general fatigue were reported by female (MFI-20, *General Fatigue*: *M* = 18.0, *SD* = 2.4) compared with male (MFI-20, *General Fatigue*: *M* = 15.7, *SD* = 3.4) participants although the range of scores on this subscale indicate individual variability across both groups. Mean scores for both sexes suggest ‘severe’ fatigue as indicated by scores above 13 [67]. (Possible scores on the MFI-20 range from 4 to 20 [56], and healthy controls average 6.8 on this subscale (see [67]). The mean years since diagnosis of ME/CFS was approximately 10 years for the total sample, female and male participants. The majority of participants (39/44) adhered to the treatment protocol (self-reported taking > 90% of the combined antibiotic and probiotic intervention).

**Table 2:** Baseline demographics for intention-to-treat sample stratified by sex

Demographics	Total sample (*N* = 44)			Females (*n* = 27)			Males (*n* = 17)		
	n	M (SD)^a^	Range	n	M (SD)^a^	Range	n	M (SD)^a^	Range
Age (years)	44	44.1 (13.5)	18–65	27	43.8 (12.8)	19–62	17	44.5 (14.9)	18–65
Hours worked per week (not parenting)	37	13.0 (16.8)	0–45	23	9.4 (15.5)	0–40	14	19.0 (17.7)	0–45
Fatigue severity (general fatigue)^b^	42	17.2 (3.0)	9–20	27	18.0 (2.4)	11–20	15	15.7 (3.4)	9–20
Duration of illness (years)	35	9.9 (6.9)	.5–26	23	9.6 (7.1)	.5–26	12	10.4 (6.6)	1–20
Frequency short duration (≤ 3 years)		7 (15.9%)			5 (18.5)			2 (11.8%)	
Frequency long duration (> 3 years)		28 (63.6%)			18 (66.7%)			10 (58.8%)	
Unknown		9 (20.45%)			4 (14.81%)			5 (29.41%)	

Information about the duration of the illness was also described by classifying into two groups of shorter (≤ 3 years) and longer (> 3 years) duration using the same ranges as in Hornig et al. [89]

^a^Data reflects *mean* (*standard deviation*) or *frequency (%)*

^b^Multidimensional Fatigue Inventory-20 (MFI-20) general fatigue subscale scores range from 4 to 20 [56] with severe fatigue indicated by scores ≥ 13 in a prior study [67]

### Outcomes and estimation

**Fig. 2:**
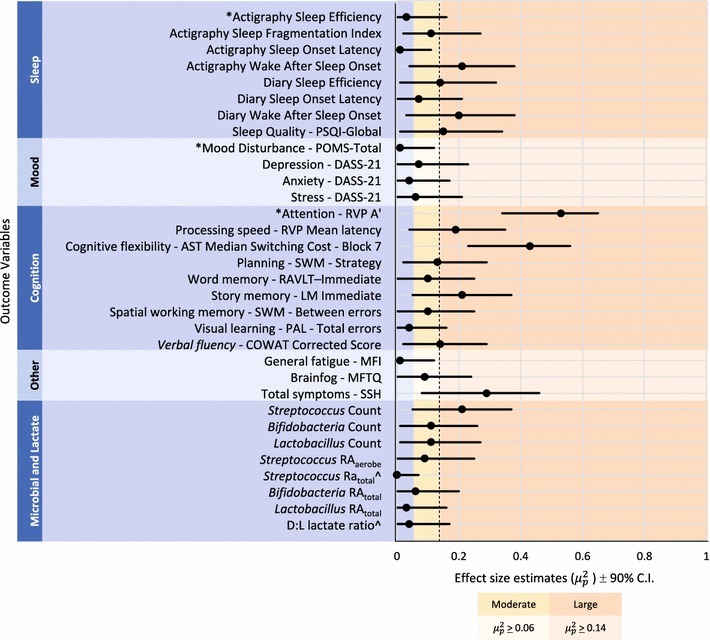
Effect size estimates ($\mu_{p}^{2}$ = partial eta squared) and confidence intervals (C.I.) for clinical, microbial and lactate outcomes for the total sample across time. The cut-off for large effects ($\mu_{p}^{2}$ > .14) is indicated by the dotted line. Asterisks (*) are used to identify primary outcomes. Change in mean scores for all clinical outcomes (sleep, mood, cognitive and other) were in the direction of improvement at post-intervention. Change in mean scores on microbiota variables reduced at post-intervention unless indicated (^). The d:l lactate variable ratio increased at post-intervention (^). See Additional file 1: Table S1 for baseline and post descriptive statistics for each variable

Descriptive results, ES estimates and exact significance levels obtained from 2 × 2 ANOVAs are presented for the total ITT sample and stratified by sex (Additional file 1: Table S1). Some outcomes had missing data due to incomplete responses (questionnaires), collection error (stool and urine samples), and/or technical error (actigraphy). Management procedures for missing and ambiguous data are presented in the Additional file 1: Additional Method. Analysis of the change in scores from baseline to post for male and female subgroups (sex-time interactions) revealed no large effects and thus did not support a sex-specific response to the treatment ($\mu_{p}^{2}$ < .014 for each outcome variable, see Additional file 1: Table S1). However, several dependent variables revealed a change across the intervention (i.e., time effects) when considering the sample as a whole. Figure 2 shows the ES estimates and confidence intervals for each outcome variable for the ITT sample.

The primary outcome for sleep, actigraphic sleep efficiency, revealed similar mean scores at baseline (*M* = 83.94, *SD* = 10.95) and post (*M* = 83.80, *SD* = 9.85) with a small effect estimate ($\mu_{p}^{2}$ = .03, *p* < .297) indicating no change in objective measurement of sleep efficiency. However, small improvements in perceived (Diary) sleep efficiency ($\mu_{p}^{2}$ = .14, *p* = .035) and sleep quality (PSQI: $\mu_{p}^{2}$ = .15, *p* = .027) were shown. There was also a reduction in awakenings during the night with approximately 20% of the between-subjects variance accounted for by the intervention/time (actigraphy WASO: $\mu_{p}^{2}$ = .21, *p* = .004; diary WASO: $\mu_{p}^{2}$ = .20, *p* = .007).

Results for primary and secondary mood outcomes indicated minimal change in group mean scores across time. The primary mood outcome, POMS total score, revealed the lowest ES estimate ($\mu_{p}^{2}$ = .01, *p* = .649) compared with DASS subscales (depression: $\mu_{p}^{2}$ = .07, *p* = .009; anxiety: $\mu_{p}^{2}$ = .04, *p* = .221; stress: $\mu_{p}^{2}$ = .06, *p* = .151).

Five of the nine cognitive outcome variables revealed large ES estimates. The primary cognitive outcome, RVP A’, suggested an improvement in sustained attention from baseline (*M* = .91, *SD* = .42) to post (*M* = .94, *SD* = .04) with 53% of the between-subjects variance accounted for by the intervention/time ($\mu_{p}^{2}$ = .53, *p* < .001). Secondary cognitive outcomes also indicated improvement across time in processing speed ($\mu_{p}^{2}$ = .19, *p* = .004), cognitive flexibility ($\mu_{p}^{2}$ = .43, *p* < .001), story memory (*μ*^2^ = .21, *p* = .002), and verbal fluency ($\mu_{p}^{2}$ = .14, *p* = .014).

The final clinical variable that suggested improvement was self-reported total symptoms (SSH) with group means reducing from baseline (*M* = 28.42, *SD* = 9.93) to post (*M* = 22.76, *SD* = 9.81) and approximately 29% of the between-subject variance attributed to the intervention/time ($\mu_{p}^{2}$ = .29, *p* = .001). Notably, large sex effects were also observed for this variable with females (*M* = 31.14, *SD* = 8.16) reporting worse total symptoms compared to males (*M* = 23.00, *SD* = 11. 27) at baseline ($\mu_{p}^{2}$ = .18, *p* = .015).

**Fig. 3:**
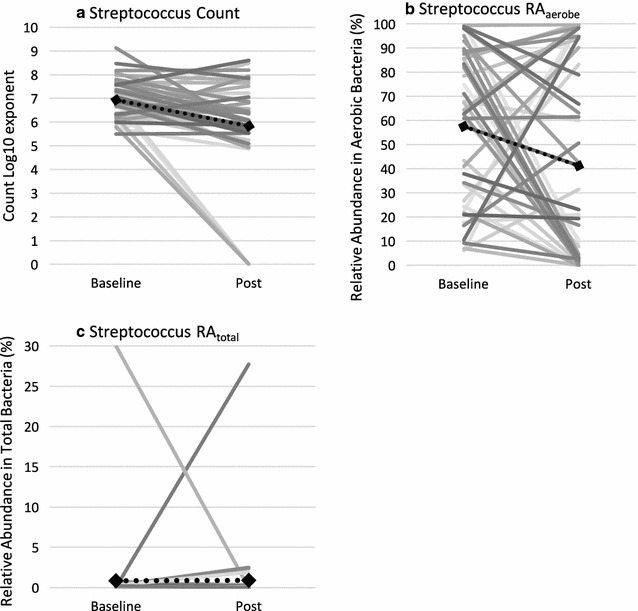
Change in *Streptococcus* (**a**) count, (**b**) relative abundance within aerobic bacteria (RA_aerobe_), and (**c**) relative abundance within total bacteria (RA_total_) for individual cases before and after intervention.  indicates *mean* scores at baseline and post

*Streptococcus* count was the only microbial variable that showed a large effect for time ($\mu_{p}^{2}$ = .21, *p* = .003) with a reduction from baseline (*M* = 8.69 × 10^6^, *SD* = 6.39) to post (*M* = 6.88 × 10^5^, *SD* = 1.39 × 10^2^). No interaction, time or sex effects were observed on the d-lactate outcome variable. Interestingly split-plot graphs of *Streptococcus* count (Fig. 3a), RA_aerobe_ (Fig. 3b), and RA_total_ (Fig. 3c) showed a spread of individual responses to the treatment with several participants increasing at post (count = 12/42, RA_aerobe_ = 17/42, RA_total_ = 13/42). In addition to this individual variability, accurate interpretation of results from ITT analyses were limited by no placebo control and the possibility of practice effects on cognitive outcomes. To better understand associations between bacterial change and symptom expression the ancillary exploratory analyses were performed.

### Ancillary exploratory analyses: correlations

Results of non-parametric correlations of variable change scores for the total sample, males and females are presented in Additional file 1: Tables S3–S5. Detailed examination of the breadth of information provided by these ancillary analyses are beyond the scope of this paper. For the purposes of this article, only correlations with large effect sizes (*r*_*s*_ > .5) are discussed to avoid over-interpretation with smaller samples and the risk of Type 1 error with multiple correlations. There were no large correlations between change in microbiota and clinical symptoms for the total sample (Additional file 1: Table S3). For females, results showed negative correlations (i.e., increased bacteria associated with clinical improvement) between change in: *Clostridium* and cognitive flexibility (*r*_*s*_ = − .58, *p* = .002), *Lactobacillus* and planning (*r*_*s*_ = −.50, *p* = .010), and *Enterococcus* and story memory (*r*_*s*_ = −.50, *p* = .015; Additional file 1: Table S4). The majority of large correlations were shown for males (see Additional file 1: Table S5). Table 3 provides a summary of large correlations between change in clinical symptoms and microbial and lactate change variables in males.

**Table 3:** Summary of large spearman’s rho (*r*_*s*_) correlations (> .5) between clinical change and microbial or lactate change variables in males

Microbial count and lactate change	Clinical change	*r*_*s*_	*p*	*n*	Direction of change in bacteria associated with clinical improvement
*Bacteroides*	Sleep quality—PSQI	− .758	.011	10	Increase
	General fatigue—MFI	− .738	.004	13	Increase
	Stress—DASS	− .701	.011	12	Increase
	Total symptoms—SSH	− .600	.067	10	Increase
	Mood disturbance—POMS total	− .573	.066	11	Increase
	Actigraphy wake after sleep onset	− .529	.043	15	Increase
	Planning—SWM—strategy	.588	.017	16	Decrease
*Bifidobacterium*	Sleep quality—PSQI	− .681	.030	10	Increase
	General fatigue—MFI	− .602	.030	13	Increase
	Anxiety—DASS	− .567	.043	13	Increase
	Visual learning—PAL—total errors	− .529	.035	16	Increase
*Clostridium*	Total symptoms—SSH	.582	.078	10	Decrease
	Verbal fluency—COWAT corrected score^a^	.550	.027	16	Decrease
	Story memory—LM immediate^a^	.522	.038	16	Decrease
	Processing speed—RVP mean latency	.507	.045	16	Decrease
*Enterococcus*	General fatigue—MFI	− .516	.071	13	Increase
*Lactobacillus*	Actigraphy sleep onset latency	.610	.016	15	Decrease
	Attention—RVP A′^a^	− .571	.021	16	Increase
	Actigraphy sleep efficiency^a^	.512	.051	15	Decrease
*Streptococcus*	Diary sleep onset latency	− .656	.006	16	Increase
d:l Lactate ratio	Actigraphy sleep onset latency	.802	.001	14	Decrease
	Total symptoms—SSH	.567	.112	9	Decrease
	General fatigue—MFI	.532	.061	13	Decrease
	Mood disturbance—POMS total	.509	.110	11	Decrease

N.B. Lower scores on clinical outcomes = improvement

^a^Variables with reversed correlations (i.e., multiplied by − 1) to allow for consistent interpretation

The correlations presented in Table 3 indicate some consistency across several clinical outcomes for the genera *Bacteroides, Bifidobacterium, Clostridium* and d:l Lactate variables. Negative correlations suggest that an increase in *Bacteroides* (as observed in 11/16 males) was associated with improvements in sleep (Actigraphy WASO, Sleep Quality—PSQI), mood (Mood Disturbance—POMS Total; Stress—DASS), general fatigue (MFI-GF) and total symptoms (SSH). An association in the opposite direction was found for change on the cognitive measure of planning (SWM-Strategy), which was reduced.

Negative correlations were shown between change in *Bifidobacterium* and sleep quality (PSQI), general fatigue (MFI), anxiety (DASS), and visual learning. Alternatively, positive correlations were revealed between change in *Clostridium* and total symptoms (SSH) and some cognitive outcomes (verbal fluency, story memory, processing speed). Notably, change in *Streptococcus* correlated negatively with perceived sleep onset (Diary SOL) indicating that reduced *Streptococcus* was associated with subjectively longer time taken to fall asleep in males.

### d:l Lactate

A small, negative correlation was observed between change in d:l lactate concentration ratios and change in *Streptococcus* count for the total sample (*r*_*s*_ = − .243, *p* = .142). Correlations with clinical symptoms revealed that the change in d:l lactate concentration ratios was positively associated with change in sleep onset latency (actigraphy SOL), mood disturbance (POMS total) general fatigue (MFI) and total symptoms (SSH) in males. This would suggest proportionally higher concentrations of d-lactate were associated with adverse symptoms in males. Proportionally higher concentrations of d-lactate were seen in 9/15 males and 12/23 females at post intervention.

### Harms

Six unexpected adverse events were reported from five participants. One participant (a) experienced severe diarrhoea, vomiting and cramping after taking the first antibiotic. This participant also experienced a respiratory allergic reaction to a non-protocol medication taken to attempt to relieve the gastrointestinal symptoms. Four other participants experienced an adverse event including (b) blood in stool (bloating but no pain reported), (c) difficulty sleeping, (d) rash on torso, and (e) exacerbation of Seborrheic dermatitis. Of these participants, the first (a) discontinued all treatment after the first antibiotic dose. The other participants (b) completed the treatment protocol, (c) reduced antibiotics (consumed 20/24 capsules), or reduced probiotics (d: consumed 11/28 capsules, e: consumed 14/28 capsules), respectively. All participants participated in post-intervention assessments.

## Discussion

ITT analysis of effects across outcome variables showed reduction in *Streptococcus* count and improvement across multiple clinical outcomes with no clear sex difference in treatment effect. The clinical changes observed with this short intervention included large effects likely to reflect modest clinical improvement on some secondary sleep outcomes (wakefulness, efficiency, quality), primary and secondary cognitive outcomes (attention, processing speed, cognitive flexibility, story memory, verbal fluency) and total symptoms. Measures of mood, fatigue and d-lactate showed no (or low) treatment effects.

Improvement on some sleep and cognitive measures appear promising considering this short intervention (4-weeks) and the complexity of this chronic condition (average illness duration ~ 10 years). It is unclear whether clinical changes at post were a direct response to the treatment or better explained by placebo, practice effects (particularly cognitive outcomes) or symptom variability of unknown origin. If placebo effects are the primary explanation for the results observed, we would have predicted consistent improvements across subjective variables (i.e., sleep, mood and fatigue variables) that were not shown. With these confounding factors in mind, improvement on objective sleep parameters may provide the most reliable indicator of change. Using these conservative parameters, reduced wakefulness after sleep onset (actigraphic WASO) may be the best indicator of clinical improvement.

Unexpectedly, individual variability of treatment response was highlighted by the proportion of participants who increased in *Streptococcus* counts at post (count = 28%, RA_aerobe_ = 41%, RA_total_ = 31%). This prompted exploration of relationships between change in microbial count and clinical symptoms. Ancillary results showed that shifts in microbiota were associated with more of the variance in clinical changes for males compared with females. Smaller correlations for females may (i) suggest non-monotonic relationships, (ii) raise questions about the benefits of the intervention for this group, (iii) suggest that other unmeasured factors may contribute to the variance observed (i.e., changes in the microbiome, hormonal, immune, other stressors) or (iv) indicate an alternate mode of action in females (i.e., not revealed by the methods carried out in this pilot study).

In males, change in *Bacteroides*, *Bifidobacterium* and *Clostridium* were associated with change across several symptoms. Intercorrelations between change in microbial and clinical variables suggest that an increase in *Bacteroides* (count) was associated with improvement on some clinical measures of sleep, mood, fatigue and total symptoms. Similarly, increased *Bifidobacterium* was associated with improvement in sleep quality, general fatigue, anxiety and visual learning. For *Clostridium*, a reduction was associated with more clinical improvements (cognitive and total symptoms).

Previous findings suggest that it would be premature to conclude that these genera are only relevant for males with ME/CFS [26, 28, 68]. Armstrong et al. [68] found reduced frequency of *Bacteroides* and increased frequency of *Clostridium* in female ME/CFS patients compared with controls. Decreased *Bacteroides* spp. in ME/CFS compared with controls and positive associations with serum amino acids [68] may be particularly relevant considering the role of amino acids for cellular energy [69]. Nagy-Szakal et al. [26] also found reduced proportion of *Bacteroides vulgatus* but an increased abundance of ‘unclassified’ *Bacteroides* using sequencing techniques in ME/CFS patients without IBS symptoms. Prior evidence combined with our results raise questions about the abundance, diversity and functional role of *Bacteroides* in ME/CFS. Therefore, a more reasonable explanation for our ancillary results may be related to observed changes in our sample. For example, a larger proportion of males (11/16, 68.8%) increased in *Bacteroides* count at post compared with females (10/26, 38.5%). Rather than pointing to sex differences as a primary factor relevant for treatment response, our results could merely reflect individual variability or could imply increased complexity in females (i.e., the influence of other confounding factors such as hormonal shifts that may account for a larger percentage of the variance).

The growth in *Bacteroides* species at post for 11/16 males may have occurred from cross-feeding through probiotic supplementation. Metabolic by-products from one bacteria can become a food source (i.e., prebiotic) for other commensal bacteria [70]. Several *Bifidobacteria* species produce complex carbohydrates (exopolysaccharides) that can become substrates for other bacteria and subsequently promote their growth [70]. Some strains of *Bifidobacterium* have been shown to increase species of *Bacteroides* using culture methods ex vivo [70, 71]. Whilst the strains analysed in prior studies are not directly comparable to the strains administered in this study (*B. lactis*, *B. breve*, *B. longum*), the possibility of similar metabolic processes should be considered. Our increasing understanding of cross-feeding and microbial communication (see review [33]) may be useful to identify probiotic or prebiotic treatment alternatives to restore microbial homeostasis.

### Relevance for d-lactate theory

The results of ITT outcome and ancillary analyses showing no change in d:l lactate ratio at post and small negative correlations between change in d:l lactate and *Streptococcus,* raise doubts about d-lactate metabolism from *Streptococcal* species. Considering, 21/38 participants increased in d:l lactate ratio after the intervention, it appears that the reduction of *Streptococcus* did not decrease d-lactate concentrations as expected. Given the enteric microbiota consists of more than 1000 species of bacteria [33], the limitations with culture-based identification methods, and the uncertainty around which species are producing lactate, it is possible that a reduction in *Streptococcus* may have allowed another d-lactate producing organism to proliferate. Some ancillary results provide partial support for d-lactate theory in males with change scores indicating decrease of d:l lactate ratio associated with improvement on some clinical outcomes (sleep onset (actigraphy SOL), mood disturbance (POMS), general fatigue (MFI), and total symptoms (SSH)). Perhaps our results reflect the relative change in reduced l-lactate production that would impact the ratio measured. Further research is needed to compare d-lactate concentrations (optimally in urine, faecal and serum samples) in ME/CFS with healthy controls and investigate other possible d-lactate producing bacteria, to adequately evaluate the relevance of the d-lactate hypothesis for either sex.

### Limitations

Our interpretation of d:l lactate is restricted by methodological limitations requiring the use of a lactate ratio. The routine use of creatinine for normalising urinary metabolites [72] may be inappropriate considering findings of higher creatinine concentrations in ME/CFS patients compared with controls [50]. Without an appropriate method for normalisation, absolute d-lactate concentrations and absolute l-lactate concentrations could not be statistically analysed because of the known wide variation in the concentration of spot urine samples in contrast to 24 h timed collections used to calculate daily excretion rates. Similarly, using genera rather than species data for microbial outcomes has reduced specificity and restricts interpretation.

The open-label design without placebo-control and using repeated measures carries inherent limitations restricting interpretation and generalisability of findings. Whilst the placebo response appears to be lower in ME/CFS than other medical conditions (e.g., depression, migraine, gastro-intestinal conditions), the influence of participant expectation appears to be greater for interventions with physiological targets (i.e., infectious or immunological) compared with psychosocial interventions in ME/CFS [73]. Discrepancies between cognitive measures and other symptoms raise questions about the influence of practice effects inherent in repeated testing over a short interval. Whilst alternate forms and outcomes with reduced practice effects were prioritised (see Additional file 1: Additional Method), ideally, controlled comparison can be used in future research to ascertain the proportion of change that can be attributed to familiarity with cognitive tests.

Other confounding factors included the influence of diet, concurrent medication and fluctuating symptomatology. Whilst we attempted to control for these factors by asking participants to remain stable on their diet and medication, the possibility of effects from other treatments or dietary intake cannot be excluded. The nature of the condition is that it has symptomatology that can be exacerbated or diminished without clear attributional cause. These fluctuations and other environmental (change in education or employment status, family stressors) and/or physiological (e.g., stage of menstrual cycle, viral/bacterial exposure) factors could not be controlled.

Statistical limitations include reduced power with smaller male samples, consideration of multiplicity of analyses and restricted interpretation with correlations. Results from correlational data only provide information about monotonic relationships, cannot attribute causation and have limited capacity to infer direct treatment effects. Cautious interpretations have been made focusing on large effects to attempt to reduce bias and improve generalisability. However, this conservative approach excludes small and moderate correlations that may also be relevant.

### Other modes of action

Some lactate results that contradict d-lactate theory prompt consideration of whether *Streptococcus* spp. or the intervention could have other modes of action. Streptococcal throat infections have been proposed as precipitating encephalitis and neurological symptoms in childhood (see [74–76]). Evidence of abnormal basal ganglia imaging and antibasal ganglia antibodies suggests that streptococcal infections may trigger autoimmune responses in some individuals [76]. Within the context of ME/CFS, it seems reasonable to explore whether the overgrowth of commensal enteric *Streptococcus*, as observed in 58/92 (59.2%) patients screened, may exert immunological or autoimmune effects that contribute to neurological symptoms. Future research could also evaluate a history of streptococci infections and monitor immune and inflammatory markers to establish whether similar mechanisms are at play in ME/CFS. Monitoring immune and inflammatory markers could be particularly beneficial considering antibiotic macrolides have immune-modulating properties that may be a mechanism responsible for improvement in this clinical sample (see [77]).

Another possible mechanism of the intervention is through the prokinetic qualities of erythromycin. Erythromycin is a macrolide that inhibits protein synthesis in specific bacteria [78] and can increase gastric motility [79]. Low doses of erythromycin have been used for its prokinetic qualities in patients with delayed gastric emptying [80]. The stimulation of oesophageal, gastric and small intestinal contractions are likely to partially explain commonly reported gastrointestinal side effects (i.e., diarrhoea, nausea, vomiting) of oral erythromycin (see [81]). Therefore, the prokinetic effect of erythromycin may be particularly beneficial for this sample when we consider that constipation is a common symptom for patients with comorbid IBS and/or small intestinal bacterial overgrowth (SIBO; [82]), and the prevalence of intestinal permeability in ME/CFS [20, 21]. Increased monitoring of gastrointestinal changes, SIBO and IBS symptoms would be useful in further studies.

Probiotics may also increase bowel transit [83] or have other modes of action. Possible mechanisms of probiotics include modulating inflammatory and immune responses through enhancing the epithelial barrier, adherence to the mucosal wall, direct (antimicrobial) or indirect (competitive exclusion) effects on pathogenic microbiota, and vagal signalling (see [33, 84–86]). Metabolic by-products from specific bacterial strains may also effect clinical presentations through the production of neurotransmitters (see [87]), short chain fatty acids through fermentation (see [33]), and cross-feeding, as discussed above. Advances in metabolomics methods would be useful to monitor functional changes during probiotic supplementation in ME/CFS patients.

## Conclusions

These results add to the accumulating evidence that microbiota–gut–brain interactions play a role in the clinical presentations of a subgroup of ME/CFS patients. This antimicrobial and probiotic treatment showed concurrent reduction in enteric *Streptococcus* counts and improvement in some neurological symptoms for the ITT sample. Precise mechanisms remain to be determined because results for d-lactate challenged the premise that *Streptococcus* species are the primary producers of d-lactic acid. Other mechanisms including the immune-modulating properties of macrolides and probiotics could be explored.

Ancillary results infer that shifts in microbiota were associated with more of the variance in clinical changes for males compared with females. It is unclear whether the reduction in *Streptococcus* is particularly beneficial in some ME/CFS patients or whether other concurrent microbial shifts are equally or more valuable (i.e., reduced *Bacteroides* and/or increased *Clostridium*). Analysis of the microbiome through sequencing techniques should be examined to elucidate other microbial shifts not revealed through culture-based methods before pursuing a randomised placebo controlled trial. Whilst sex differences were not obvious through primary analyses, ancillary results reinforce the need to recruit sufficient samples to enable sex comparisons in clinical trials.

Individual differences in microbial and clinical changes observed across this intervention are unsurprising considering other prominent findings in gut microbiome and ME/CFS research. For example, ground-breaking research with a large healthy cohort has shown the microbiome as a primary predictor of varied glucose response to the same foods, supporting the need for personalised nutrition [88]. Within ME/CFS, duration of illness [89] and genetic variability [90–92] appear to be key factors that contribute to differences in immune markers, pathophysiology and clinical presentation. Considering the bidirectional role of the gut microbiome in immune modulation (e.g., [93]), epigenetic regulation [94], and the influence of genetics on microbial composition [95], continued efforts to understand the function of the microbiome in ME/CFS is warranted.

## Additional files

**Additional file 1.** Supplementary method and tables S1, S2, S7–S9

**Additional file 2.** Supplementary tables S3–S6

## Abbreviations

ANOVA: analysis of variance
CANTAB: Cambridge Neuropsychological Test Automated Battery
CONSORT: consolidated standards of reporting trials
DASS: Depression Anxiety Stress Scale
EES: erythromycin ethyl succinate
ES: effect sizes
IBS: irritable bowel syndrome
ITT: intention to treat
MALDI-TOF-MS: Matrix Assisted Laser Absorption and Ionisation Time of Flight Mass Spectrometry
MANOVA: multiple analysis of variance
ME/CFS: myalgic encephalomyelitis/chronic fatigue syndrome
MFI: Multidimensional Fatigue Inventory
POMS: Profile of Mood States-Short Form
PSQI: Pittsburgh Sleep Quality Index
RA: relative abundance
RVP: Rapid Visual Attention
SE: sleep efficiency
SFI: sleep fragmentation index
SIBO: small intestinal bacterial overgrowth
SOL: sleep onset latency
SPSS: Statistical Package for the Social Sciences
SSH: Symptom Severity and Symptom Hierarchy Profile
SWM: Short-term Working Memory
WASO: wake after sleep onset

## References

[1] Carruthers BM, van de Sande MI, De Meirleir KL, Klimas NG, Broderick G, Mitchell T (2011). Myalgic encephalomyelitis: international consensus criteria. J Intern Med.

[2] Jason LA, Brown A, Clyne E, Bartgis L, Evans M, Brown M (2012). Contrasting case definitions for chronic fatigue syndrome, myalgic encephalomyelitis/chronic fatigue syndrome and myalgic encephalomyelitis. Eval Health Prof.

[3] Jason LA, Evans M, Brown A, Sunnquist M, Newton JL (2015). Chronic fatigue syndrome versus sudden onset myalgic encephalomyelitis. J Prev Interv Community.

[4] Clayton EW (2015). Beyond myalgic encephalomyelitis/chronic fatigue syndrome: an IOM report on redefining an illness. JAMA.

[5] Buchwald D, Umali P, Umali J, Kith P, Pearlman T, Komaroff AL (1995). Chronic fatigue and the chronic fatigue syndrome: prevalence in a Pacific northwest health care system. Ann Intern Med.

[6] Steele L, Dobbins JG, Fukuda K, Reyes M, Randall B, Koppelman M (1998). The epidemiology of chronic fatigue in San Francisco. Am J Med.

[7] Reyes M, Nisenbaum R, Hoaglin DC, Unger ER, Emmons C, Randall B (2003). Prevalence and incidence of chronic fatigue syndrome in Wichita, Kansas. Arch Intern Med.

[8] Reeves WC, Jones JF, Maloney E, Heim C, Hoaglin DC, Boneva RS (2007). Prevalence of chronic fatigue syndrome in metropolitan, urban, and rural Georgia. Popul Health Metr.

[9] Lawrie SM, Manders DN, Geddes JR, Pelosi AJ (1997). A population-based incidence study of chronic fatigue. Psychol Med.

[10] Nacul LC, Lacerda EM, Pheby D, Campion P, Molokhia M, Fayyaz S (2011). Prevalence of myalgic encephalomyelitis/chronic fatigue syndrome (ME/CFS) in three regions of England: a repeated cross-sectional study in primary care. BMC Med.

[11] Fuhrer R, Wesseley S (1995). The epidemiology of fatigue and depression: a French primary-care study. Psychol Med.

[12] Jason L, Brown M, Evans M, Anderson V, Lerch A, Brown A (2011). Measuring substantial reductions in functioning in patients with chronic fatigue syndrome. Disabil Rehabil.

[13] Anderson VR, Jason LA, Hlavaty LE, Porter N, Cudia J (2012). A review and meta-synthesis of qualitative studies on myalgic encephalomyelitis/chronic fatigue syndrome. Patient Educ Couns.

[14] Marshall R, Paul L, Wood L (2011). The search for pain relief in people with chronic fatigue syndrome: a descriptive study. Physiother Theory Pract.

[15] Ware NC (1998). Sociosomatics and illness in chronic fatigue syndrome. Psychosom Med.

[16] Jason LA, Benton MC, Valentine L, Johnson A, Torres-Harding S (2008). The economic impact of ME/CFS: individual and societal costs. Dyn Med.

[17] Friedberg F, Jason L (1998). Understanding chronic fatigue syndrome: an empirical guide to assessment and treatment.

[18] Aaron LA, Burke MM, Buchwald D (2000). Overlapping conditions among patients with chronic fatigue syndrome, fibromyalgia, and temporomandibular disorder. Arch Intern Med.

[19] Chia JK, Chia AY (2007). Chronic fatigue syndrome is associated with chronic enterovirus infection of the stomach. J Clin Pathol.

[20] Maes M, Leunis JC (2008). Normalization of leaky gut in chronic fatigue syndrome (CFS) is accompanied by a clinical improvement: effects of age, duration of illness and the translocation of LPS from gram-negative bacteria. Neuroendocrinol Lett.

[21] Maes M, Twisk FN, Kubera M, Ringel K, Leunis JC, Geffard M (2012). Increased IgA responses to the LPS of commensal bacteria is associated with inflammation and activation of cell-mediated immunity in chronic fatigue syndrome. J Affect Disord.

[22] Butt HL, Dunstan RH, McGregor NR, Roberts TK, Harrison TL, Granger JR. Faecal microbial growth inhibition in chronic fatigue/pain patients. In: Proceedings of the AHMF International Clinical and Scientific Conference. Sydney, Australia; 1998.

[23] Sheedy JR, Wettenhall REH, Ssanlon D, Gooley PR, Lewis DP, McGregor NR (2009). Increased d-lactic acid intestinal bacteria in patients with chronic fatigue syndrome. In Vivo (Brooklyn).

[24] Frémont M, Coomans D, Massart S, De Meirleir K (2013). High-throughput 16S rRNA gene sequencing reveals alterations of intestinal microbiota in myalgic encephalomyelitis/chronic fatigue syndrome patients. Anaerobe.

[25] Giloteaux L, Goodrich JK, Walters WA, Levine SM, Ley RE, Hanson MR (2016). Reduced diversity and altered composition of the gut microbiome in individuals with myalgic encephalomyelitis/chronic fatigue syndrome. Microbiome.

[26] Nagy-Szakal D, Williams BL, Mishra N, Che X, Lee B, Bateman L (2017). Fecal metagenomic profiles in subgroups of patients with myalgic encephalomyelitis/chronic fatigue syndrome. Microbiome.

[27] Jackson ML, Butt H, Ball M, Lewis DP, Bruck D (2015). Sleep quality and the treatment of intestinal microbiota imbalance in chronic fatigue syndrome: a pilot study. Sleep Sci.

[28] Wallis A, Butt H, Ball M, Lewis DP, Bruck D. Support for the microgenderome: associations in a human clinical population. Sci Rep. 2016; 6:19171. 10.1038/srep19171 PMC4725945 26757840

[29] Wallis A, Butt H, Ball M, Lewis DP, Bruck D (2017). Support for the microgenderome invites enquiry into sex differences. Gut Microbes.

[30] Cryan JF, Dinan TG (2012). Mind-altering microorganisms: the impact of the gut microbiota on brain and behaviour. Nat Rev Neurosci.

[31] Rhee SH, Pothoulakis C, Mayer EA (2009). Principles and clinical implications of the brain-gut-enteric microbiota axis..

[32] Mayer EA (2011). Gut feelings: the emerging biology of gut-brain communication. Nat Rev Neurosci.

[33] Ohland CL, Jobin C (2015). Microbial activities and intestinal homeostasis: a delicate balance between health and disease. Cell Mol Gastroenterol Hepatol.

[34] Bested AC, Logan AC, Selhub EM (2013). Intestinal microbiota, probiotics and mental health: from Metchnikoff to modern advances: part II—contemporary contextual research. Gut Pathog.

[35] Bailey MT, Coe CL (1999). Maternal separaseparation disrupts the integrity of the intestinal microflora in infant rhesus monkeys. Dev Psychobiol.

[36] Desbonnet L, Garrett L, Clarke G, Kiely B, Cryan JF, Dinan TG (2010). Cognitive, behavioral, and systems neuroscience: effects of the probiotic *Bifidobacterium infantis* in the maternal separation model of depression. Neuroscience.

[37] O’Mahony SM, Marchesi JR, Scully P, Codling C, Ceolho AM, Quigley EMM (2009). Early life stress alters behavior, immunity, and microbiota in rats: implications for irritable bowel syndrome and psychiatric illnesses. Biol Psychiatry.

[38] Sullivan A, Nord CE, Evengård B (2009). Effect of supplement with lactic-acid producing bacteria on fatigue and physical activity in patients with chronic fatigue syndrome. Nutr J.

[39] Rao AV, Bested AC, Beaulne TM, Katzman MA, Iorio C, Berardi JM (2009). A randomized, double-blind, placebo-controlled pilot study of a probiotic in emotional symptoms of chronic fatigue syndrome. Gut Pathog.

[40] Groeger D, O’Mahony L, Murphy EF, Bourke JF, Dinan TG, Kiely B (2013). Bifidobacterium infantis 35624 modulates host inflammatory processes beyond the gut. Gut Microbes.

[41] Borody TJ, Nowak A, Finlayson S (2012). The GI microbiome and its role in chronic fatigue syndrome: a summary of bacteriotherapy. J Australas Coll Nutr Environ Med.

[42] Wallis A, Ball M, McKechnie S, Butt H, Lewis DP, Bruck D (2017). Examining clinical similarities between myalgic encephalomyelitis/chronic fatigue syndrome and d-lactic acidosis: a systematic review. J Transl Med.

[43] Tappenden KA (2014). Pathophysiology of short bowel syndrome: considerations of resected and residual anatomy. JPEN J Parenter Enteral Nutr.

[44] Petersen C (2005). d-Lactic acidosis. Nutr Clin Pract Off Publ Am Soc Parenter Enter Nutr.

[45] Goffin P, Deghorain M, Mainardi JL, Tytgat I, Champomier-Vergès MC, Kleerebezem M (2005). Lactate racemization as a rescue pathway for supplying d-lactate to the cell wall biosynthesis machinery in *Lactobacillus plantarum*. J Bacteriol.

[46] Flak MB, Neves JF, Blumberg RS (2013). Welcome to the microgenderome. Science (80-).

[47] Carruthers BM, Jain AK, DeMeirleir KL, Peterson DL, Klimas NG, Lerner AM (2003). Myalgic encephalomyelitis/chronic fatigue syndrome: clinical working case definition, diagnostic and treatment protocols. J Chronic Fatigue Syndr.

[48] Therapeutic Goods Administration. The Australian Clinical Trial Handbook: simple, practical guide to the conduct of clinical trials to International standards of Good Clinical Practice (GCP) in the Australian context. Australian Government, Department of Health and Ageing; 2006.

[49] Scheijen JLJM, Hanssen NMJ, Van De Waarenburg MPH, Stehouwer CDA, Schalkwijk CG, Jonkers DMAE (2012). L(+) and D(−) lactate are increased in plasma and urine samples of type 2 diabetes as measured by a simultaneous quantification of L(+) and D(−) lactate by reversed-phase liquid chromatography tandem mass spectrometry. Exp Diabetes Res.

[50] Armstrong CW, McGregor NR, Lewis DP, Butt HL, Gooley PR (2015). Metabolic profiling reveals anomalous energy metabolism and oxidative stress pathways in chronic fatigue syndrome patients. Metabolomics.

[51] Coull JT, Middleton HC, Robbins TW, Sahakian BJ (1995). Clonidine and diazepam have differential effects on tests of attention and learning. Psychopharmacology.

[52] Curran SL, Others A (1995). Short form of the profile of mood states (POMS-SF): psychometric Information. Psychol Assess.

[53] Cambridge Cognition. CANTAB [Cognitive assessment software]. 2015.

[54] Buysse DJ, Reynolds CF, Monk TH, Berman SR, Kupfer DJ, CFR CF (1989). The Pittsburgh Sleep Quality Index: a new instrument for psychiatric practice and research. Psychiatry Res.

[55] Lovibond SH, Lovibond PF (1995). Manual for the depression anxiety stress scales.

[56] Smets EM, Garssen B, Bonke B, De Haes JC (1995). The multidimensional fatigue inventory (MFI) psychometric qualities of an instrument to assess fatigue. J Psychosom Res.

[57] Jason LA, Jessen T, Porter N, Boulton A, Gloria-Njoku M, Friedberg F (2009). Examining types of fatigue among individuals with ME/CFS. Disabil Stud Q.

[58] IBM Corp. IBM SPSS Statistics for Windows.

[59] Moher D, Hopewell S, Schulz KF, Montori V, Gøtzsche PC, Devereaux PJ (2012). CONSORT 2010 explanation and elaboration: updated guidelines for reporting parallel group randomised trials. Int J Surg.

[60] Cohen J (1988). Statistical power analysis for the behavioral sciences.

[61] Wuensch KL (2016). Using SPSS to obtain a confidence interval for R-squared from regression.

[62] Feng C, Wang H, Lu N, Chen T, He H, Lu Y (2014). Log-transformation and its implications for data analysis. Shanghai Arch Psychiatry.

[63] Altman DG (1999). Practical statistics for medical research.

[64] Pallant JF (2016). SPSS survival manual: a step by step guide to data analysis using IBM SPSS.

[65] Tabachnick BG, Fidell LS (2013). Using multivariate statistics.

[66] Fritz CO, Morris PE, Richler JJ (2012). Effect size estimates: current use, calculations, and interpretation. J Exp Psychol Gen.

[67] Majer M, Welberg LA, Capuron L, Miller AH, Pagnoni G, Reeves WC (2008). Neuropsychological performance in persons with chronic fatigue syndrome: results from a population-based study. Psychosom Med.

[68] Armstrong CW, Gooley PR, McGregor NR, Lewis DP, Butt HL (2017). The association of fecal microbiota and fecal, blood serum and urine metabolites in myalgic encephalomyelitis/chronic fatigue syndrome. Metabolomics.

[69] Da Poian AT, El-Bacha T, Luz MRMP (2010). Nutrient utilization in humans: metabolism pathways. Nat Educ.

[70] Salazar N, Gueimonde M, Hernández-Barranco AM, Ruas-madiedo P, Clara G (2008). Exopolysaccharides produced by intestinal Bifidobacterium strains act as fermentable substrates for human intestinal bacteria. Appl Environ Microbiol.

[71] Rios-Covian D, Cuesta I, Alvarez-Buylla JR, Ruas-Madiedo P, Gueimonde M, de los Reyes-Gavilán CG (2016). Bacteroides fragilis metabolises exopolysaccharides produced by bifidobacteria. BMC Microbiol.

[72] Barr D, Wilder L, Caudill S, Gonzalez A, Needham L, Pirkle J (2005). Urinary creatinine concentrations in the U.S. population: implications for urinary biologic monitoring measurements. Environ Health Perspect.

[73] Cho HJ, Hotopf M, Wessely S (2005). The placebo response in the treatment of chronic fatigue syndrome: a systematic review and meta-analysis. Psychosom Med.

[74] Swedo SE, Leonard HL, Garvey M, Mittleman B, Allen AJ, Perlmutter S (1998). Pediatric autoimmune neuropsychiatric disorders associated with streptococcal infections: clinical description of the first 50 cases. Am J Psychiatry.

[75] Swedo S, Seidlitz J, Kovacevic M, Latimer M, Hommer R, Lougee L (2015). Clinical presentation of pediatric autoimmune neuropsychiatric disorders associated with streptococcal infections in research and community settings. J Child Adolesc Psychopharmacol.

[76] Dale RC, Neville BG, Goddard E, Cox TC, Kling Chong WK, Williams A (2001). Poststreptococcal acute disseminated encephalomyelitis with basal ganglia involvement and auto-reactive antibasal ganglia antibodies. Ann Neurol.

[77] Vermeulen RCW, Scholte HR (2006). Azithromycin in chronic fatigue syndrome (CFS), an analysis of clinical data. J Transl Med.

[78] Wolfe A, Hahn F (1964). Erythromycin: mode of action. Science (80-).

[79] Itoh Z, Nakaya M, Suzuki T, Arai H, Wakabayashi K (1984). Erythromycin mimics exogenous motilin in gastrointestinal contractile activity in the dog. Am J Physiol.

[80] Sanger GJ, Broad J, Andrews PLR (2013). Perspective: the relationship between gastric motility and nausea: gastric prokinetic agents as treatments. Eur J Pharmacol.

[81] Curry JI, Lander TD, Stringer MD (2001). Erythromycin as a prokinetic agent in infants and children. Aliment Pharmacol Ther.

[82] Pimentel M, Chow EJ, Lin HC (2000). Eradication of small intestinal bacterial overgrowth reduces symptoms of irritable bowel syndrome. Am J Gastroenterol.

[83] Spiller R (2008). Review article: probiotics and prebiotics in irritable bowel syndrome. Aliment Pharmacol Ther.

[84] Bermudez-Brito M, Plaza-Diaz J, Munoz-Quezada S, Gomez-Llorente C, Gil A (2012). Probiotic mechanisms of action. Ann Nutr Metab.

[85] Thomas CM, Versalovic J (2010). Probiotics-host communication: modulation of signaling pathways in the intestine. Gut Microbes.

[86] Wang Y, Kasper LH (2014). The role of microbiome in central nervous system disorders. Brain Behav Immun.

[87] O’Mahony SM, Clarke G, Borre YE, Dinan TG, Cryan JF (2015). Serotonin, tryptophan metabolism and the brain-gut-microbiome axis. Behav Brain Res.

[88] Zeevi D, Korem T, Zmora N, Israeli D, Rothschild D, Weinberger A (2015). Personalized nutrition by prediction of glycemic responses. Cell.

[89] Hornig M, Montoya JG, Klimas NG, Levine S, Felsenstein D, Bateman L (2015). Distinct plasma immune signatures in ME/CFS are present early in the course of illness. Sci Adv.

[90] Billing-Ross P, Germain A, Ye K, Keinan A, Gu Z, Hanson MR (2016). Mitochondrial DNA variants correlate with symptoms in myalgic encephalomyelitis/chronic fatigue syndrome. J Transl Med.

[91] Johnston S, Staines D, Klein A, Marshall-Gradisnik S (2016). A targeted genome association study examining transient receptor potential ion channels, acetylcholine receptors, and adrenergic receptors in chronic fatigue syndrome/myalgic encephalomyelitis. BMC Med Genet.

[92] Schlauch KA, Khaiboullina SF, De Meirleir KL, Rawat S, Petereit J, Rizvanov AA (2016). Genome-wide association analysis identifies genetic variations in subjects with myalgic encephalomyelitis/chronic fatigue syndrome. Transl Psychiatry.

[93] Markle JGM, Frank DN, Mortin-Toth S, Robertson CE, Feazel LM, Rolle-Kampczyk U (2013). Sex differences in the gut microbiome drive hormone-dependent regulation of autoimmunity. Science (80-).

[94] Lee E, Song E, Nam Y (2017). Dysbiosis of gut microbiome and its impact on epigenetic regulation. J Clin Epigenet.

[95] Goodrich JK, Waters JL, Poole AC, Sutter JL, Koren O, Blekhman R (2014). Human genetics shape the gut microbiome. Cell.

