# Correction to: Open-label pilot for treatment targeting gut dysbiosis in myalgic encephalomyelitis/chronic fatigue syndrome: neuropsychological symptoms and sex comparisons

**DOI:** 10.1186/s12967-018-1413-y

**Published:** 2018-02-23

**Authors:** Amy Wallis, Michelle Ball, Henry Butt, Donald P. Lewis, Sandra McKechnie, Phillip Paull, Amber Jaa‑Kwee, Dorothy Bruck

**Affiliations:** 1 Psychology Department, College of Health and Biomedicine, Victoria University, Melbourne, Australia; 2 Bioscreen (Aust) Pty Ltd., Melbourne, Australia; 3 CFS Discovery Clinic, Donvale, Melbourne, Australia; 4 College of Engineering and Science, Victoria University, Melbourne, Australia

## Correction to: J Transl Med (2018) 16:24 10.1186/s12967-018-1392-z

The original version of this article [1], published on 6 February 2018, contains a mistake in the ‘Conclusions’ section. The corrected version of the affected sentence is given below and the corrected part is marked in bold.

- It is unclear whether the reduction in *Streptococcus* is particularly beneficial in some ME/CFS patients or whether other concurrent microbial shifts are equally or more valuable **(i.e., increased** ***Bacteroides*** **and/or reduced** ***Clostridium*)**.
