# Supplementary material for: Open-label pilot for treatment targeting gut dysbiosis in myalgic encephalomyelitis/chronic fatigue syndrome: neuropsychological symptoms and sex comparisons
Source: J Transl Med. 2018 Feb 6;16:24. doi: 10.1186/s12967-018-1392-z (PMC5801817; doi:10.1186/s12967-018-1392-z)
Supplement: Supplementary file 1 — Additional file 1. Supplementary method and tables S1, S2, S7–S9 [file 12967_2018_1392_MOESM1_ESM.docx]

**ADDITIONAL MATERIAL**

**Open-label pilot for treatment targeting gut dysbiosis in myalgic encephalomyelitis/chronic fatigue syndrome: Neuropsychological symptoms and sex comparisons**

Amy Wallis^1^, Michelle Ball^1^, Henry Butt^2^, Donald P. Lewis^3^, Sandra McKechnie^4^, Phillip Paull^2^, Amber Jaa-Kwee^4^, Dorothy Bruck^1^

^1^Psychology Department, College of Health and Biomedicine, Victoria University, Melbourne, Australia

^2^Bioscreen (Aust) Pty Ltd., Melbourne, Australia

^3^CFS Discovery Clinic, Donvale, Melbourne, Australia

^4^College of Engineering and Science, Victoria University, Melbourne, Australia

**This PDF File Includes:**

Supplementary method

Tables S1-2, S7-S9

**ADDITIONAL METHOD**

This supplementary material provides detailed information about clinical, microbial and lactate measures and procedures used during the trial. Operational definitions of sleep terminology, measurement methods, and procedures for managing ambiguous and missing data are presented to aid replication. The rationale for selected outcome variables is provided with reference made to Spearman’s rho correlations between baseline clinical outcome variables (see Table S6).

**Measuring Sleep Symptoms**

**Objective Sleep - Actigraphy**

**Overview and considerations**

Actigraphy was used as an objective measure of sleep patterns in this sample. Actigraphy instruments provide a non-invasive measure of sleep/wake behavior through analysis of activity and light intensity. Actigraphy methods have been shown to reliably estimate sleep and wake patterns for several clinical populations across the lifespan [1]. The use of actigraphy within ME/CFS populations has high ecological validity [2] and has been previously employed [3, 4].

Actigraphy has high sensitivity but low specificity. Intervention studies have shown that actigraphy is sensitive to measuring change after both pharmacological and psychological interventions (see [5]). Sensitivity measurement of sleep onset latency (SOL), wake after sleep onset (WASO) and sleep efficiency (SE) variables seem to vary dependent on the clinical population being examined (see Table S7 for operational definitions of sleep terminology). The primary methodological issue relates to the device’s low specificity or accuracy when detecting wakefulness during a sleep period [5]. This has been repeatedly indicated for the measurement of SOL and beckons cautious interpretation of this outcome variable [6]. Low specificity is likely to partially explain inconsistencies between some subjective and objective sleep parameters.

Actigraphy data provides information about movement (rather than sleep) that is then used as an indicator of sleep/wake states [5]. Therefore, the data could be influenced by neurobehavioural or motor system disorders [5]. Whilst these factors influence the reliability and validity of actigraphy assessment, with the available technology, actigraphy provides an unobtrusive form of assessing sleep patterns within the home setting [7] and measuring treatment efficacy [5]. Compensatory measures (i.e., recording duration over 5 days; use of established scoring protocols) and adjunctive measures (i.e., concurrent completion of a sleep diary) were used to help overcome some limitations of actigraphy devices [1, 5–7].

**Participant procedures**

Participants wore the Actiwatch monitors (Respironics Actiware 2) on their non-dominant hand for 7 consecutive days/nights during baseline and post-intervention weeks. Participants were asked to press the silver button on the actiwatch (to signal an ‘event marker’ on the data) to indicate when they attempted to fall asleep for the night and at final awakening. Participants were encouraged to leave the watch on for the 7 day period and attempt to keep the watch uncovered during the day to prevent disruption to light sensor data. To reduce variability between Actiwatch monitors, the same watch was used for baseline and post-intervention data collection when possible.

**Data Scoring Protocol**

Procedures outlined by the Society of Behavioral Sleep Medicine were followed to score sleep and wake periods [1]. Sleep/wake patterns during the main sleep interval were used to obtain objective outcome measures. Actiware 6.08 default analysis properties for 30 second epoch lengths were used to calculate sleep parameters (*Sleep* = activity counts < 40; *Wake* = activity counts > 40). Sleep onset and final awakening were determined by 10 immobile minutes (see Table S7 for definitions of sleep terminology).

The Actiware program automatically predicted the main sleep interval by inserting a *rest* interval. However, this default *rest* interval did not always accurately reflect the main sleep interval. The extended bed rest and lowered activity observed in this population frequently resulted in ambiguous start and endpoints and inaccurate placement of the *rest* interval. As a common issue in many sleep disorders and other medical conditions [1], the following decision hierarchy was used to determine the start (*lights out*) and end (*rise* i.e., the time the participant got out of bed for the day) of the main sleep period in accordance with the recommended guidelines (see [1]).

1. All automatic *‘rest’* intervals were screened to ensure they met the following criteria:
   1. Sleep period start time coincided with
      1. a marked decrease in light
      2. a marked decrease in activity
      3. event marker signaling that the participant pressed the button to indicate attempting to fall asleep (if used)
   2. Sleep period end time (rise) coincided with:
      1. an increase in light
      2. an increase in activity
      3. event marker signaling that the participant pressed the button to indicate final awakening (if used)
2. In the event of discrepancies between any of the above conditions, a *manual* *‘rest’* interval was inserted with the following conditions considered in order of priority:
   1. Sleep start time:
      1. decrease in light
      2. decrease in activity
      3. event marker
      4. sleep diary – time the participant turned off the lights and attempted to fall asleep
   2. Sleep period end time (*rise*):
      1. increase in activity
      2. increase in light
      3. sleep diary *rise* time.
         1. In the event that the participant noted that they had remained in bed due to symptoms of the illness, the *final awakening* time was used as the end of the sleep period.
      4. event marker
3. All incomplete main sleep intervals were omitted from analysis. The occurrences of this were when:
   - 1. The watch was removed and forgotten to be replaced prior to sleep.
     2. Data was not recorded due to technical errors or watch removal that lasted for longer than 1 hour during the main sleep interval.

**Possible outcome variables**

Default algorithms were used to determine approximate values for *total sleep time* (TST), *SOL,* *WASO,* *wake bouts* (WB), *SE* and *sleep fragmentation index* (SFI). See Table S7 for an explanation of terminology and methods of measurement.

**Subjective Sleep**

**Sleep diary**

***Overview and considerations***

Objective measures of sleep complement, rather than replace, subjective sleep assessment. Sleep diaries have been routinely used and proclaimed as the ‘gold standard’ method of measuring subjective reports of sleep in healthy and clinical populations [8]. Whilst adding to participant burden and confounded by expectation bias, sleep diaries provide an advantage over objective measures when considering the individual experience of sleep and cognitive-affective factors impacting the sleep experience [9]. This is particularly pertinent considering the discrepancies that have been shown between subjective and actigraphic assessment of sleep in both healthy and clinical populations (e.g., [10, 11]) and specifically in ME/CFS samples (see [2]). Within ME/CFS, comparison between sleep diary and objective measures (polysomnography and actigraphy) suggest moderate to high consistency between methods, particularly for TST, SE and WASO [9]. Additionally, the accuracy of sleep diary data as a measurement of SOL appears preferable to actigraphy [9].

***Sleep diary material and procedure***

Selected items from the standardized protocol procedures for sleep diaries (see [8]) were incorporated in the sleep diary for this study. Relevant items were chosen to gather information about sleep time, quality, wakefulness and sleep-related behavior.

During baseline (7 nights) and post-intervention (7 nights), participants completed the sleep diary each morning in relation to the previous night’s sleep. Participants reported the use of any prescription or non-prescription sleep aids (including alcohol). Participants noted the time they a) got into bed, b) turned off the lights to fall asleep (*lights out*), c) of their final awakening, and d) got out of bed for the day (*rise*). They also indicated the number of minutes it took them to fall asleep (SOL), and the number (WB) and duration of awakenings (WASO) during the night. On two separate 5-point Likert scales participants rated sleep quality (1 = very poor, 5 = very good) and how rested or refreshed they felt when they awoke (1 = not at all rested, 5 = very well-rested).

***Possible outcome variables***

Subjective sleep parameters were operationally defined to avoid construct confusion and for the purpose of replication (see Table S7). Possible outcomes included TST, SOL, WASO, WB, SE, and *duration of sleep episode* (DSE). Subjective measures are referred to as ‘Diary’ to distinguish between actigraphic measures. SE is frequently calculated in sleep disorder research and clinical practice to reflect difficulties with falling asleep or staying asleep as indicated by the ratio between *total sleep time* (TST) to *time in bed* (TIB; [12]). Prior research has used inconsistent and ill-defined methods to determine TIB resulting in recent recommendations to clarify terminology and the suggestion of using *duration of sleep episode* (DSE = SOL + TST + WASO + *time attempting to sleep after final awakening*: TASAFA) as the denominator in the SE equation (see [12]). Sleep diary data in this study did not obtain a measurement of TASAFA because participants were not asked to describe their intentions between their *final awakening* (FA) and the *time they got out of bed for the day* (Rise). Considering other ME/CFS symptoms could affect the length of time participants spent in bed in the morning, FA was used to indicate the end of the sleep period.

- - - SE was calculated as TST/ DSE x 100, with higher percentages indicating more efficient sleep.
    - When there was missing data for SOL and WASO, DSE was calculated by the time between *lights out* and FA in minutes.

**Sleep questionnaires**

Two scales were used to measure sleep quality (*Pittsburgh Sleep Quality Index,* PSQI, [13]) and sleep disturbance (*Insomnia Sleep Index*, ISI, [14]). The PSQI has high reliability and validity as a brief measure (10 items) of perceived sleep quality with the rater reflecting on their sleep habits over the previous month [13]. The PSQI has been used to quantify non-restorative sleep in ME/CFS populations [15, 16]. Some alterations to the PSQI were made to suit our study design. Firstly, participants were instructed to rate their sleep habits based on the ‘past 2 weeks’ to increase specificity of post-intervention ratings. Item 10 was also removed from this scale. This item requires completion by a ‘bed partner’ and answers are not included when calculating the Global Score. The PSQI Global Score was calculated following instructions by [13] with scores ranging between 0-21 and lower scores indicating better sleep quality. Normative data on the PSQI from the original validation study using US samples with ‘healthy’ controls aged 24-83 years (n = 52) indicated that a Global PSQI scores greater than 5 is indicative of “poor” sleep quality [13].

The ISI is a 7-item, 5-point Likert scale that provides subjective information about the nature and severity of insomnia symptoms and impact on the individual's functioning [14, 17]. The Patient version was selected to enable self-administration with total scores ranging between 0 and 28. Comparison with sleep diary data suggests that the ISI has adequate internal consistency, albeit lower correlations with sleep diary variables indicative of insomnia symptoms (i.e., SOL, WASO; [17]). The ISI has validity for use as an outcome measure in treatment research [18].

**Selected Outcome Variables for Sleep**

The methods used to obtain information about sleep symptoms resulted in numerous variables that needed to be reduced to aid interpretation. Table S7 provides an explanation of sleep parameters and the rationale for retaining or excluding measures of sleep through actigraphic and sleep diary assessment. Baseline correlations with all clinical symptoms (see Table S6) and prior research helped form these decisions. Factor analysis methods were considered but not employed due to small sample size, inadequate case:variable ratio and considering intercorrelations < .3 between several sleep variables [19]. Therefore, selected sleep outcomes included: Actigraphy SOL, WASO, SE, SFI; Diary SOL, WASO, SE; and PSQI Global Score. The PSQI was retained as a measure of sleep quality and the unique contribution suggested by intercorrelations. The ISI was excluded due to intercorrelations with the PSQI, mood and fatigue variables that could be difficult to distinguish the unique contribution of this scale. Diary SOL and WASO were considered sufficient measures of self-reported insomnia symptoms.

**Measuring Mood Symptoms**

**Profile of Mood States**

The Profile of Mood States (POMS) Short Form is a list of 37 adjectives asking participants to rate current mood states on a 5-point Likert scale (*Not at all* = 0 to *Extremely* = 4; [20]). POMS clusters adjectives into 6 factors: tension/anxiety, depression/dejection, anger/hostility, fatigue/inertia, vigour/activity, and confusion/bewilderment. The POMS Total Mood Disturbance score is calculated from the sum of all negative clusters and subtraction of the Vigour/Activity cluster. Possible scores range from -24 to 148 with lower scores indicative of less mood disturbance. The POMS provides a measure of psychological distress [20] and appears to be useful as a treatment sensitive measure for this population [4].

Participants were asked to rate their mood on Days 7 and 42 of the study, based on their experiences ‘over the past week including today’. The POMS Total Mood Disturbance score was selected as the primary outcome variable a priori.

**Depression Anxiety Stress Scale**

The Depression Anxiety Stress Scale (DASS-21; [21]) was selected as a psychometrically sound non-diagnostic measure of self-reported symptoms of anxiety, depression and stress. Developed from the original 48-item scale [21], this shorter version is frequently used in clinical and research settings for its ease of administration, brevity and sensitivity to treatment change. The DASS-21 has Australian normative data [22] and moderate-strong psychometric properties observed in clinical [23] and nonclinical populations [24].

This 21-item scale asks raters to indicate their agreement to statements based on their experience over the past week using a 4-point likert scale (*Did not apply to me at all* = 0 to *Applied to me very much, or most of the time* = 3). Seven items pertain to each of the three subscales (DASS-Depression, DASS-Anxiety, DASS-Stress) with maximum scores of 21 indicating more distress on each dimension respectively. The DASS-21 has high reliability and discriminant validity supporting the three-factor structure in this scale [25]. It is frequently employed in clinical and nonclinical populations in both clinical and research settings [26]. Please note that the DASS-21 manual suggests doubling total scores, however, this study chose to use the raw subscale scores to be consistent with the Australian validation study [22].

**Mood Adjectives Checklist**

The Mood Adjectives Checklist (MAC; [27]) was chosen as a daily rating of positive and negative mood states to be completed during Baseline and Post-intervention weeks. The MAC has adequate reliability and validity as a daily measure of mood [27] and evidence of being able to separate positive (MAC-Positive) and negative affect as two independent subscales [28]. The extended scale was employed with 13 adjectives aligned with positive affect (happy, joyful, enjoyment/fun, pleased, energetic, relaxed, alert) and negative affect (depressed/blue, unhappy, angry/hostile, frustrated, worried/anxious, fatigued) (Porter et al., 2000). Participants rated their current mood on a 7-point Likert scale (*Not at all* = 0, *Extremely* = 7). Daily scores were calculated for MAC-Positive (higher scores, more positive) and MAC-Negative (higher scores, more negative) subscales. Mean weekly scores on each subscale were calculated from daily scores.

**Selected Outcome Variables for Mood**

Baseline intercorrelations between POMS subscale and total scores, MAC factors and the DASS subscales suggested overlapping measurement of similar dimensions (see Table S6). Baseline intercorrelations between the POMS Total Mood Disturbance and other POMS subscales were moderate to strong (*r* = .65 to .86). Therefore, the POMS Total Mood Disturbance score was considered representative of POMS subscale scores. To reduce the number of variables for analysis, the POMS Total Mood Disturbance score (primary outcome) and DASS subscales (DASS-Depression, DASS-Anxiety and DASS-Stress) were selected for further analysis. The POMS measure was prioritized compared with the MAC factors because of a priori selection as a primary outcome variable measuring mood.

**Measuring Cognitive Symptoms**

**Cognitive Test Battery**

Seven standardized tests were selected to measure attention, memory, verbal fluency, inhibition and planning. Table S8 provides a summary of the skills assessed by each test, administration information and selected outcome variables. These tests were chosen after evaluating psychometric properties, suitability for use with ME/CFS patients, length of administration, cost and availability.

Alternate forms were used when available to reduce practice effects (see Table S8). The use of alternate forms has been shown to reduce practice effects on the RAVLT and COWAT [29]. Parallel forms (Form A and B) of the test battery were counterbalanced using random allocation to reduce possible differences in the level of difficulty that could interfere with treatment effects [30]. Outcome variables with reduced practice effects were prioritized.

**Participant procedures**

The total administration time for the test battery was approximately 60 minutes, with 90 minutes allocated to allow for sufficient rest period between testing. Test administrators noted the length and activity type during each rest interval at baseline testing to allow for replication at post-intervention. A touchscreen laptop was used to administer the 4 tests from the Cambridge Neuropsychological Test Automated Battery (CANTAB [31]) and the remaining tests were delivered orally. Standardized test conditions and the order of tests remained consistent for baseline and post-intervention sessions. Before commencing the trial, participants selected their preferred location (either CFS Discovery Clinic or Victoria University campuses) to conduct the sessions during baseline (Day 1) and post-intervention (chosen day during week 6).

**Selected Outcome Variables for Cognitive Symptoms**

Selected outcome variables and the corresponding rationale for inclusion are summarized in Table S8.

**Measuring Other ME/CFS Symptoms**

**Total ME/CFS Symptoms**

The *Symptom Severity and Symptom Hierarchy Profile* (SSH; [32]) was used as an indicator of total ME/CFS symptoms. The scale provides a list of ME/CFS symptoms and asks respondents to rate their severity of symptoms (*Absent* = 0 to *Severe* = 3) and rank their three most severe symptoms according to their experience over the past week. This symptom profile was developed to aid diagnosis and treatment in accordance with the ME/CFS Clinical Working Case Definition [32]. Total scores are weighted based on the severity of ratings (summed responses are multiplied: absent (x0) mild (x1), moderate (x2) and severe (x3)). Higher scores indicate more severe symptoms.

**Selected outcome variable for global symptoms**

The total score was selected as an outcome measure that is indicative of overall frequency and severity of ME/CFS symptoms (*Total Symptoms-SSH*).

**Fatigue**

The Multidimensional Fatigue Inventory (MFI-20) was originally validated with ME/CFS patients to assesses fatigue across five dimensions (General Fatigue, Physical Fatigue, Motivation Level, Activity Level and Mental Fatigue [33]). Using a 5-point likert scale (*yes, that is true* = 1 to *no, that is not true* = 5) participants were asked to indicate their level of agreement with each statement considering how they have felt ‘over the past 7 days’. The MFI-20 has shown support for a 5-factor model, good internal consistency, test-retest reliability, construct and convergent validity (see [34, 35]). This scale has been used in multiple international studies with evidence for treatment sensitivity and use as a primary outcome measure for ME/CFS (e.g., [36]). Items were scored according to instructions by Smets, Garson and Bonke [37] with higher scores indicating more fatigue. Scores for each subscale range from 4 to 20 with higher scores indicating greater fatigue.

The *Brain Fog* subscale of the Multiple Fatigue Types Questionnaire (MTFQ, [38]) was used as a measure of ‘brain fog’ that is a common symptom of ME/CFS related to mental fatigue/exhaustion and associated disruption to thinking, attention, processing and memory [38]. This measure was specifically developed to assess fatigue in individuals with ME/CFS. As a new scale, it has adequate internal reliability and substantiates the notion of cognitive fatigue distinct from other fatigue types [38]. MFTQ items were scored on the same 7-point likert scale as the MAC items to reduce confusion because these items were presented together within the Participant Response Booklet. Scores from the three items of the MFTQ were summed as an indicator of daily cognitive fatigue (*Brain Fog* subscale range of scores: 3-21). A weekly mean score was calculated from daily ratings for the MFTQ-Brainfog outcome variable.

**Selected outcome variables for fatigue**

With the primary focus on sleep, mood and cognitive symptoms in this current paper, only two outcome measures were chosen for fatigue. The authors of the MFI-20 recommend using the General Fatigue subscale (MFI-GF) as a preferred global estimation of fatigue rather than summation of all subscales [37]. Therefore, MFI-GF was selected as a secondary outcome to represent general/global fatigue in participants in this study. The MFTQ-Brainfog mean weekly score (Brainfog-MFTQ) was selected as another fatigue outcome variable due to low correlations with MFI-GF at baseline (*r_s_ =* .061*, p* = .701) and other MFI subscales (see Table S6).

**Microbiota and Lactate Measurement**

During screening and post-intervention phases, participants were asked to collect their first morning stool and mid-stream urine samples (after 5am) independently in their own home. Participants were provided with detailed instructions of how to collect a mid-stream urine sample to avoid cross-contamination with bacteria from other sources. Participants were asked to refrain from food and beverages from 10pm on the night prior to collection. After passing the first portion (5-10mL) of urine, a sample of 5-10mL of urine was collected in a sterile specimen container, stored in a zip lock bag and stored in the fridge. Both urine and stool samples were collected by courier and transported in cold conditions (<12 °C) to Bioscreen laboratory within 48 hours after sample collection.

Methods of faecal collection, transportation and identification of microbiota using MALDI-TOF MS analysis were the same as those described in [39]. Urinary lactate concentrations were determined using High Performance Liquid Chromatography and Triple Quadrupole Mass Spectrometry (HPLC-TMS) as outlined in the main article.

**Microbial Outcome Variables**

Three genera were prioritised for analysis of microbial outcomes based on treatment target (*Streptococcus*) and probiotic supplementation (*Bifidobacterium* and *Lactobacillus*). Both count and relative abundance (RA) variables were used to examine change in frequency and proportion of each genus. RA_total_ was calculated by the ratio of each genus count divided by total detectable bacteria count. The proportion of *Streptococcus* within total aerobic bacteria (RA_aerobe_) was also used as an outcome measure to be consistent with inclusion criteria and aid clinical interpretation.

**Lactate Outcome Variable**

Higher concentrations of creatinine have been shown in ME/CFS compared with controls [40]. Therefore, routine normalisation with creatinine [41] was deemed inappropriate and restricted use of absolute D-lactate concentrations and absolute L-lactate concentrations. Hence, the D:L lactate concentrations ratio was used as a secondary outcome variable.

**Procedure for Handling Missing or Ambiguous Data**

Table S9 provides a summary of the scoring procedures used for handling missing and ambiguous data on self-report measures and faecal microbial analysis.

**REFERENCES**

1. Ancoli-Israel S, Martin JL, Blackwell T, Buenaver L, Liu L, Meltzer LJ, et al. The SBSM Guide to Actigraphy Monitoring: Clinical and Research Applications. Behav Sleep Med. 2015;13:S4–38.

2. Jackson ML, Bruck D. Sleep abnormalities in chronic fatigue syndrome/myalgic encephalomyelitis: a review. J Clin Sleep Med. 2012;8:719–28.

3. Tajima S, Kuratsune H, Yamaguti K, Takahashi A, Takashima S, Watanabe Y, et al. Estimation of fatigue state in patient with CFS using actigraph and R-R interval power spectrum analysis. Japanese J Clin Med. 2007;65:1057–64.

4. Jackson ML, Butt H, Ball M, Lewis DP, Bruck D. Sleep quality and the treatment of intestinal microbiota imbalance in Chronic Fatigue Syndrome: A pilot study. Sleep Sci. 2015;8:124–33.

5. Sadeh A. Clinical Review: The role and validity of actigraphy in sleep medicine: An update. Sleep Med Rev. 2011;15:259–67.

6. Martin, JL, Hakim, AD. Wrist actigraphy. Chest. 2011;139:1514–27.

7. Ustinov Y, Lichstein KL. Actigraphy Reliability with Normal Sleepers. Behav Sleep Med. 2013;11:313–20.

8. Carney CE, Buysse DJ, Ancoli-Israel S, Edinger JD, Krystal AD, Lichstein KL, et al. The consensus sleep diary: standardizing prospective sleep self-monitoring. Sleep. 2012;35:287–302.

9. Creti L, Libman E, Baltzan M, Rizzo D, Bailes S, Fichten CS. Impaired sleep in Chronic Fatigue Syndrome. J Health Psychol. 2010;15:596–607.

10. Kobayashi I, Lavela J, Mellman TA, Huntley E. Subjectively and objectively measured sleep with and without posttraumatic stress disorder and trauma exposure. Sleep. 2012;35:957–65.

11. Wang M-Y, Hung H-L, Tsai P-S. The sleep log and actigraphy: congruency of measurement results for heart failure patients. J Nurs Res (Lippincott Williams Wilkins). 2011;19:173–80.

12. Reed DL, Sacco WP. Measuring Sleep Efficiency: What Should the Denominator Be? J Clin Sleep Med JCSM Off Publ Am Acad Sleep Med. 2016;12:263–6.

13. Buysse DJ, Reynolds CF, Monk TH, Berman SR, Kupfer DJ, III CFR, et al. The Pittsburgh Sleep Quality Index: a new instrument for psychiatric practice and research. Psychiatry Res. 1989;28:193–213.

14. Morin CM. Insomnia : psychological assessment and management. New York : Guilford Press; 1993.

15. Neu D, Mairesse O, Hoffmann G, Dris A, Lambrecht LJ, Linkowski P, et al. Sleep quality perception in the chronic fatigue syndrome: correlations with sleep efficiency, affective symptoms and intensity of fatigue. Neuropsychobiology. 2007;56:40–6.

16. Neu D, Mairesse O, Verbanck P, Le Bon O. Slow wave sleep in the chronically fatigued: Power spectra distribution patterns in chronic fatigue syndrome and primary insomnia. Clin Neurophysiol. 2015.

17. Bastien CH, Vallières A, Morin CM. Original article: Validation of the Insomnia Severity Index as an outcome measure for insomnia research. Sleep Med. 2001;2:297–307.

18. Morin CM, Belleville G, Bélanger L, Ivers H. The Insomnia Severity Index: psychometric indicators to detect insomnia cases and evaluate treatment response. Sleep. 2011;34:601–8.

19. Pallant JF. SPSS survival manual : a step by step guide to data analysis using IBM SPSS. Sydney : Allen & Unwin, 2016.; 2016.

20. Curran SL, Others A. Short Form of the Profile of Mood States (POMS-SF): Psychometric Information. Psychol Assess. 1995;7:80–3.

21. Lovibond SH, Lovibond PF. Manual for the Depression Anxiety Stress Scales. 2nd ed. Sydney, Australia: Psychology Foundation of Australia; 1995.

22. Crawford J, Cayley C, Lovibond PF, Wilson PH, Caroline H. Percentile Norms and Accompanying Interval Estimates from an Australian General Adult Population Sample for Self-Report Mood Scales (BAI, BDI, CRSD, CES-D, DASS, DASS-21, STAI-X, STAI-Y, SRDS, and SRAS). Aust Psychol. 2011;46:3–14.

23. Page AC, Hooke GR, Morrison DL. Psychometric properties of the Depression Anxiety Stress Scales (DASS) in depressed clinical samples. Br J Clin Psychol. 2007;46 Pt 3:283–97.

24. Osman A, Wong JL, Bagge CL, Freedenthal S, Gutierrez PM, Lozano G. The Depression Anxiety Stress Scales-21 (DASS-21): Further Examination of Dimensions, Scale Reliability, and Correlates. J Clin Psychol. 2012;68:1322–38.

25. Henry JD, Crawford JR. The short-form version of the Depression Anxiety Stress Scales (DASS-21): Construct validity and normative data in a large non-clinical sample. Br J Clin Psychol. 2005;44:227–39.

26. Ronk FR, Korman JR, Hooke GR, Page AC. Assessing clinical significance of treatment outcomes using the DASS-21. Psychol Assess. 2013;25:1103–10.

27. Emmons RA, Diener E. Personality Correlates of Subjective Well-Being. Pers Soc Psychol Bull. 1985;11:89.

28. Diener E, Emmons RA. The independence of positive and negative affect. J Pers Soc Psychol. 1984;47:1105–17.

29. Beglinger LJ, Gaydos B, Tangphao-Daniels O, Duff K, Kareken DA, Crawford J, et al. Practice effects and the use of alternate forms in serial neuropsychological testing. Arch Clin Neuropsychol. 2005;20:517–29.

30. Hawkins KA, Dean D, Pearlson GD. Alternative Forms of the Rey Auditory Verbal Learning Test: A Review. Behav Neurol. 2004;15:99-107.

31. CANTAB [Cognitive assessment software]. Cambridge Cognition. 2015. All rights researved. www.cantab.com

32. Carruthers BM, Jain AK, DeMeirleir KL, Peterson DL, Klimas NG, Lerner AM, et al. Myalgic Encephalomyelitis/Chronic Fatigue Syndrome: Clinical working case definition, diagnostic and treatment protocols. J Chronic Fatigue Syndr. 2003;11:7–116.

33. Smets EM, Garssen B, Bonke B, De Haes JC. The Multidimensional Fatigue Inventory (MFI) psychometric qualities of an instrument to assess fatigue. J Psychosom Res. 1995;39:315–25.

34. Hewlett S, Dures E, Almeida C. Measures of Fatigue. Arthritis Care & Res. 2011;63:S263-S286.

35. Whitehead L. The measurement of fatigue in chronic illness: a systematic review of unidimensional and multidimensional fatigue measures. J Pain Symptom Manage. 2009;37:107–28.

36. Montoya JG, Kogelnik AM, Bhangoo MS, Lunn MR, Flamand L, Merrihew LE, et al. Randomized clinical trial to evaluate the efficacy and safety of valganciclovir in a subset of patients with chronic fatigue syndrome. J Med Virol. 2013;85:2101–9.

37. Smets E, Garssen B, Bonke B. Multidimensional Fatigue Inventory Instructions. Amsterdam: Academic Medical Center, University of Amsterdam; 2013.

38. Jason LA, Jessen T, Porter N, Boulton A, Gloria-Njoku M, Friedberg F. Examining Types of Fatigue Among Individuals with ME/CFS. Disabil Stud Q. 2009;29:9.

39. Wallis A, Butt H, Ball M, Lewis DP, Bruck D. Support for the Microgenderome: Associations in a Human Clinical Population. Sci Rep. 2016;6:19171.

40. Armstrong CW, McGregor NR, Lewis DP, Butt HL, Gooley PR. Metabolic profiling reveals anomalous energy metabolism and oxidative stress pathways in chronic fatigue syndrome patients. Metabolomics. 2015;11:1626–39.

41. Barr DB, Wilder, LC, Caudill, SP, Gonzalez, AJ, Needham, LL, Pirkle, JL. Urinary Creatinine Concentrations in the U.S. Population: Implications for Urinary Biologic Monitoring Measurements. Environ Heal Perspect. 2005;113:192.

42. Stevens C. Investigation of naturalistic sleep/wake behaviour in myalgic encephaloyelitis/chronic fatigue syndrome. 2014.

43. Jones GM, Sahakian BJ, Levy R, Warburton DM, Gray JA. Effects of acute subcutaneous nicotine on attention, information processing and short-term memory in Alzheimer’s disease. Psychopharmacology (Berl). 1992;108:485–94.

44. Cambridge Cognition. Overview of CANTAB Tests. Cambridge; 2017.

45. Morris RG, Downes JJ, Sahakian BJ, Evenden JL, Heald A, Robbins TW. Planning and spatial working memory in Parkinson’s disease. J Neurol Neurosurg Psychiatry. 1988;51:757–66.

46. Robbins TW, James M, Owen AM, Sahakian BJ, McInnes L, Rabbit P. A neural systems approach to the cognitive psychology of ageing using the CANTAB battery: Studies with CANTAB on a large sample of the normal elderly population. In: Methodology of Frontal and Executive Function. Rabbitt P, editor. United Kingdom: Psychology Press; 1997. p. 215–38.

47. Rey A. L’examen clinique en psychologie [Clinical tests in psychology]. Paris; 1964.

48. Schmidt M. Rey Auditory Verbal Learning Test: A handbook. Los Angeles: Western Psychological Services; 1996.

49. Lezak MD, Loring DW, Howieson DB. Neuropsychological assessment. Oxford : Oxford University Press; 2004.

50. Carstairs JR, Shores EA, Myors B. Australian Norms and Retest Data for the Rey Auditory and Verbal Learning Test. Aust Psychol. 2012;47:191–7.

51. Weschler D. Weschler Memory Scale-Fourth Edition. San Antonio, TX: Pearson; 2009.

52. Morris J, Woodworth C, Umfleet LG, Czipri S, Kopald B, Swier-Vosnos A. Development of alternate paragraphs for the logical memory subtest of the wechsler memory scale-IV. Appl Neuropsychol. 2014;21:143–7.

53. Benton A, Hamsher K. Multilingual Aphasia Examination manual. Iowa City: University of Iowa; 1976.

54. Strauss, EA, Spreen O, Sherman, E. A compendium of neuropsychological tests: Administration, norms and commentary. 2nd edition. New York: Oxford University Press; 2006.

**TABLES**

**Table S1.** Results of 2x2 ANOVAs for all outcome variables with descriptive statistics, effect size estimates and exact significance values

| **Outcomes** | **Total Sample** | | | **Females** | | | **Males** | | | **Sex-Time Interaction** | | **Time** | | **Sex** | |
| --- | --- | --- | --- | --- | --- | --- | --- | --- | --- | --- | --- | --- | --- | --- | --- |
|  |  | Baseline | Post |  | Baseline | Post |  | Baseline | Post |  |  |  |  |  |  |
|  | *n* | *M(SD)* | *M(SD)* | *n* | *M(SD)* | *M(SD)* | *n* | *M(SD)* | *M(SD)* | $\mu_{p}^{2}$ *(90%CI)* | *P* | $\mu_{p}^{2}$ *(90%CI)* | *P* | $\mu_{p}^{2}$ *(90%CI)* | *P* |
| ***Sleep*** |  |  |  |  |  |  |  |  |  |  |  |  |  |  |  |
| Actigraphy Sleep Efficiency^ | 38 | 82.94 (10.95) | 83.80 (9.85) | 23 | 81.94 (13.14) | 82.52 (11.68) | 15 | 84.48 (6.42) | 85.75 (5.93) | 0.00(0.00, 0.09) | 0.697 | 0.03(0.00, 0.16) | 0.297 | 0.02(0.00, 0.14) | 0.395 |
| Actigraphy Sleep Fragmentation Index* | 38 | 25.99 (11.28) | 24.46 (11.44) | 23 | 26.93 (12.97) | 25.93 (13.38) | 15 | 24.55 (8.27) | 22.20 (7.47) | 0.02(0.00, 0.14) | 0.402 | 0.11(0.02, 0.27) | 0.042 | 0.02(0.00, 0.14) | 0.416 |
| Actigraphy Sleep Onset Latency | 38 | 22.39 (19.72) | 21.23 (30.54) | 23 | 22.24 (20.57) | 23.59 (37.54) | 15 | 22.62 (19.05) | 17.61 (15.07) | 0.02(0.00, 0.14) | 0.381 | 0.01(0.00, 0.11) | 0.612 | 0.00(0.00, 0.09) | 0.723 |
| Actigraphy Wake After Sleep Onset* | 38 | 48.87 (24.75) | 43.28 (24.06) | 23 | 53.49 (27.02) | 49.85 (27.53) | 15 | 41.78 (19.54) | 33.21 (12.66) | 0.04(0.00, 0.18) | 0.228 | 0.21(0.04, 0.38) | 0.004 | 0.09(0.00, 0.25) | 0.07 |
| Diary Sleep Efficiency^ | 33 | 89.01 (5.26) | 91.74 (4.92) | 21 | 87.91 (4.72) | 91.41 (5.02) | 12 | 90.93 (5.79) | 92.31 (4.90) | 0.03(0.00, 0.17) | 0.345 | 0.14(0.01, 0.32) | 0.035 | 0.06(0.00, 0.22) | 0.188 |
| Diary Sleep Onset Latency | 44 | 30.77 (24.17) | 25.47 (23.42) | 27 | 31.56 (22.48) | 25.76 (22.63) | 17 | 29.52 (27.30) | 25.02 (25.32) | 0.00(0.00, 0.05) | 0.823 | 0.07(0.00, 0.21) | 0.082 | 0.02(0.00, 0.15) | 0.84 |
| Diary Wake After Sleep Onset | 34 | 27.55 (21.49) | 15.62 (14.19) | 22 | 31.55 (21.39) | 16.67 (12.88) | 12 | 20.20 (20.50) | 13.69 (16.75) | 0.04(0.00, 0.18) | 0.271 | 0.20(0.03, 0.38) | 0.007 | 0.05(0.00, 0.21) | 0.184 |
| Sleep Quality - PSQI-Global | 32 | 9.78 (4.24) | 8.13 (3.72) | 21 | 9.76 (4.61) | 8.10 (3.51) | 11 | 9.82 (3.63) | 8.18 (4.29) | 0.00(0.00, 0.00) | 0.983 | 0.15(0.01, 0.34) | 0.027 | 0.00(0.00, 0.00) | 0.958 |
| ***Mood*** |  |  |  |  |  |  |  |  |  |  |  |  |  |  |  |
| Mood Disturbance - POMS-Total* | 32 | 45.08 (24.11) | 43.18 (27.32) | 20 | 43.58 (22.41) | 41.79 (32.04) | 12 | 47.58 (27.56) | 45.50 (17.91) | 0.00(0.00, 0.01) | 0.972 | 0.01(0.00, 0.12) | 0.649 | 0.01(0.00, 0.11) | 0.655 |
| Depression - DASS-21 | 40 | 5.98 (4.76) | 5.00 (4.78) | 26 | 5.85 (4.81) | 5.19 (5.34) | 14 | 6.21 (4.84) | 4.64 (3.67) | 0.01(0.00, 0.12) | 0.478 | 0.07(0.00, 0.23) | 0.09 | 0.00(0.00, 0.00) | 0.951 |
| Anxiety - DASS-21 | 40 | 4.25 (3.68) | 3.63 (3.63) | 26 | 4.50 (3.44) | 4.04 (3.74) | 14 | 3.79 (4.17) | 2.86 (3.42) | 0.01(0.00, 0.09) | 0.678 | 0.04(0.00, 0.17) | 0.221 | 0.02(0.00, 0.14) | 0.386 |
| Stress - DASS-21 | 38 | 8.24 (5.36) | 6.87 (5.16) | 25 | 8.16 (5.23) | 6.68 (5.60) | 13 | 8.39 (5.81) | 7.23 (4.38) | 0.00(0.00, 0.04) | 0.857 | 0.06(0.00, 0.21) | 0.151 | 0.00(0.00, 0.06) | 0.808 |
| ***Cognition*** |  |  |  |  |  |  |  |  |  |  |  |  |  |  |  |
| *Executive functioning* |  |  |  |  |  |  |  |  |  |  |  |  |  |  |  |
| Attention - RVP A'^* | 43 | 0.91 (0.42) | 0.94 (0.04) | 26 | 0.91 (0.04) | 0.94 (0.05) | 17 | 0.91 (0.04) | 0.94 (0.03) | 0.00(0.00, 0.07) | 0.786 | 0.53(0.34, 0.65) | 0 | 0.00(0.00, 0.07) | 0.753 |
| Processing speed - RVP Mean latency* | 43 | 474.82 (116.49) | 442.21 (113.51) | 26 | 499.85 (137.73) | 454.62 (135.14) | 17 | 436.53 (58.36) | 423.23 (68.25) | 0.06(0.00, 0.21) | 0.104 | 0.19(0.04, 0.35) | 0.004 | 0.05(0.00, 0.18) | 0.173 |
| Cognitive flexibility - AST Median Switching Cost - Block 7 | 44 | 284.82 (141.35) | 213.05 (149.63) | 27 | 311.15 (147.92) | 235.28 (150.92) | 17 | 243.00 (122.94) | 177.74 (144.90) | 0.00(0.00, 0.08) | 0.677 | 0.43(0.23, 0.56) | 0 | 0.49(0.00, 0.18) | 0.149 |
| Planning - SWM - Strategy | 44 | 30.75 (7.74) | 28.65 (8.08) | 27 | 29.30 (7.05) | 27.11 (7.28) | 17 | 33.06 (8.43) | 31.12 (8.87) | 0.00(0.00, 0.02) | 0.881 | 0.13(0.02, 0.29) | 0.015 | 0.07(0.00, 0.21) | 0.093 |
| *Memory* |  |  |  |  |  |  |  |  |  |  |  |  |  |  |  |
| Word memory - RAVLT–Immediate^ | 44 | 49.66 (9.51) | 52.45 (8.58) | 27 | 52.07 (7.95) | 55.48 (6.60) | 17 | 45.82 (10.73) | 47.65 (9.33) | 0.01(0.00, 0.11) | 0.514 | 0.10(0.00, 0.25) | 0.035 | 0.18(0.04, 0.34) | 0.004 |
| Story memory - LM Immediate^ | 44 | 23.23 (7.70) | 26.68 (7.04) | 27 | 24.96 (7.07) | 28.07 (7.38) | 17 | 20.47 (8.07) | 24.47 (7.38) | 0.00(0.00, 0.08) | 0.681 | 0.21(0.05, 0.37) | 0.002 | 0.09(0.00, 0.24) | 0.044 |
| Spatial working memory - SWM - Between errors* | 44 | 22.80 (19.10) | 18.11 (18.02) | 27 | 17.93 (16.01) | 12.71 (11.38) | 17 | 30.53 (21.46) | 26.71 (23.12) | 0.00(0.00, 0.02) | 0.739 | 0.10(0.00, 0.25) | 0.036 | 0.14(0.02, 0.30) | 0.011 |
| Visual learning - PAL - Total errors | 44 | 16.07 (23.31) | 12.91 (14.16) | 27 | 14.04 (23.30 | 9.96 (11.88) | 17 | 19.29 (23.65) | 17.59 (16.48) | 0.01(0.00, 0.09) | 0.6 | 0.04(0.00, 0.16) | 0.205 | 0.03(0.00, 0.15) | 0.25 |
| *Verbal fluency -* COWAT Corrected Score^ | 44 | 36.82 (11.99) | 39.21 (8.97) | 27 | 38.93 (12.49) | 39.85 (8.51) | 17 | 33.47 (10.65) | 38.18 (9.84) | 0.07(0.00, 0.21) | 0.093 | 0.14(0.02, 0.29) | 0.014 | 0.03(0.00, 0.15) | 0.252 |
| ***Other ME/CFS Symptoms*** |  |  |  |  |  |  |  |  |  |  |  |  |  |  |  |
| General fatigue - MFI | 40 | 17.45 (2.74) | 16.88 (2.96) | 26 | 18.12 (2.39) | 16.88 (3.00) | 14 | 16.21 (2.99) | 16.86 (3.01) | 0.10(0.01, 0.30) | 0.042 | 0.01(0.00, 0.12) | 0.513 | 0.04(0.00, 0.17) | 0.247 |
| Brainfog - MFTQ | 43 | 10.52 (4.31) | 8.35 (4.10) | 27 | 10.72 (3.86) | 8.35 (4.10) | 16 | 10.19 (5.10) | 9.12 (3.11) | 0.01(0.00, 0.12) | 0.45 | 0.09(0.00, 0.24) | 0.05 | 0.00(0.00, 0.02) | 0.898 |
| Total symptoms - SSH | 33 | 28.42  (9.93) | 22.76 (9.81) | 22 | 31.14 (8.16) | 25.27 (9.22) | 11 | 23.00 (11.27) | 17.73 (9.36) | 0.00(0.00, 0.04) | 0.852 | 0.29(0.08, 0.46) | 0.001 | 0.18(0.02, 0.36) | 0.015 |
| ***Microbiota*** |  |  |  |  |  |  |  |  |  |  |  |  |  |  |  |
| *Streptococcus* Count | 42 | 8.69x10^6^ (6.39) | 6.88x10^5^ (1.39x10^2^) | 26 | 5.44x10^6^ (4.58) | 2.87x10^5^ (3.58x10^2^) |  | 1.87x10^6^  (8.39) | 2.86x10^5^  (1.04x10^1^) | 0.01(0.00, 0.12) | 0.485 | 0.21(0.05, 0.37) | 0.003 | 0.09(0.00, 0.24) | 0.053 |
| *Bifidobacteria* Count | 42 | 1.49x10^4^  (2.04x10^4^) | 5.08x10^2^ (8.05x10^3^) | 26 | 6.38x10^3^ (1.83x10^4^) | 3.90x10^2^ (9.44x10^3^) | 16 | 5.85x10^4^ (2.90x10^4^) | 7.80x10^2^ (8.17x10^3^) | 0.01(0.00, 0.09) | 0.64 | 0.11(0.01, 0.26) | 0.034 | 0.01(0.00, 0.10) | 0.574 |
| *Lactobacillus* Count* | 42 | 4.69x10^2^ (5.33x10^3^) | 1.91x10^2^ (2.12x10^3^) | 26 | 8.85x10^1^ (2.08x10^3^) | 2.45x10^2^ (2.56x10^3^) | 16 | 7.03x10^3^ (1.42x10^4^) | 1.27x10^2^ (1.96x10^3^) | 0.11(0.01, 0.27) | 0.032 | 0.11(0.01, 0.27) | 0.032 | 0.02(0.00, 0.13) | 0.425 |
| *Streptococcus* RA_aerobe_ | 42 | 57.59 (32.17) | 40.64 (43.66) | 26 | 56.74 (32.22) | 40.64 (43.66) | 16 | 58.96 (33.09) | 42.33 (37.59) | 0.00(0.00, 0.01) | 0.974 | 0.09(0.00, 0.25) | 0.048 | 0.00(0.00, 0.05) | 0.823 |
| *Streptococcus* RA_total_*^M^ | 42 | 0.86 (4.61) | 0.88 (4.29) | 26 | 0.13 (0.22) | 1.23 (5.43) | 16 | 2.05 (7.46) | 0.31 (0.75) | 0.05 (0.00, 0.18) | 0.165 | 0.00(0.00, 0.07) | 0.750 | 0.00(0.00, 0.12) | 0.620 |
| *Bifidobacteria* RA_total_ | 42 | 5.78 (14.98) | 1.96 (6.19) | 26 | 6.33 (16.92) | 2.46 (7.22) | 16 | 4.88 (11.61) | 1.16 (4.12) | 0.00(0.00, 0.00) | 0.976 | 0.06(0.00, 0.20) | 0.121 | 0.06(0.00, 0.09) | 0.625 |
| *Lactobacillus* RA_total_* | 42 | 2.59 (9.05) | 1.76 (7.36) | 26 | 0.35 (1.61) | 2.61 (9.27) | 16 | 6.24 (14.03) | 0.37 (1.29) | 0.12(0.01, 0.28) | 0.025 | 0.03(0.00, 0.16) | 0.258 | 0.03(0.00, 0.15) | 0.305 |
| ***Lactate*** |  |  |  |  |  |  |  |  |  |  |  |  |  |  |  |
| D:L lactate ratio* | 38 | 0.30 (0.21) | 0.34 (0.20) | 23 | 0.34 (0.24) | 0.34 (0.23) | 15 | 0.24 (0.18) | 0.33 (0.16) | 0.04(0.00, 0.17) | 0.247 | 0.04(0.00, 0.17) | 0.254 | 0.03(0.00, 0.16) | 0.322 |

$\mu_{p}^{2}$ = partial eta squared / effect size estimate; * = Levene's test violated, use p<.01; ^M^ = Box M test of equality of covariance violated

Lower scores reflect better symptoms for all clinical variables unless indicated by ^

Units of measurement for microbial variables: count = cfu/g back-transformed from Log10; RA_aerobe_ = proportion of genus count within total aerobic bacterial counts as a percentage; RA_total_ = relative abundance of each genus within total bacteria count (aerobic + anaerobic) presented as a percentage.

Large effect size estimates ($\mu^{2}$>0.14) are highlighted.

**Table S2.** Median and range for primary and secondary outcomes at baseline and post intervention for total sample

| **Outcomes** | **Baseline** | **Post** |
| --- | --- | --- |
|  | *Mdn[range]* | *Mdn[range]* |
| ***Sleep*** |  |  |
| Actigraphy Sleep Efficiency^ | 86.01[31.72, 94.10] | 86.64[45.94, 95.56] |
| Actigraphy Sleep Fragmentation Index | 22.17[12.33, 61.57] | 20.96[10.81, 55.93] |
| Actigraphy Sleep Onset Latency | 21.60[1.93, 98.71] | 11.18[0.71, 177.64] |
| Actigraphy Wake After Sleep Onset | 37.93[16.83, 100.71] | 34.04[15.29, 104.93] |
| Diary Sleep Efficiency^ | 88.52[79.52, 98.28] | 92.57[79.26, 98.96] |
| Diary Sleep Onset Latency | 27.93[0.36, 106.67] | 19.02[0.57, 110.00] |
| Diary Wake After Sleep Onset | 20.64[0, 85] | 9.48[0, 56] |
| Sleep Quality - PSQI-Global | 9[1, 18] | 7.5[1, 14] |
| ***Mood*** |  |  |
| Mood Disturbance - POMS-Total | 39[7, 94] | 35[-3, 105] |
| Depression - DASS-21 | 5[0, 18] | 4[0, 21] |
| Anxiety - DASS-21 | 3[0, 14] | 2[0, 13] |
| Stress - DASS-21 | 7.5[1, 20] | 6.5[0, 20] |
| ***Cognitive Outcomes*** |  |  |
| *Executive functioning* |  |  |
| Attention - RVP A'^ | 0.91[0.82, 0.99] | 0.94[0.85, 1.00] |
| Procesisng speed - RVP Mean latency | 469.26[323.00, 1044.33] | 417.22[289.00, 917.69] |
| Cognitive flexibility - AST Median Switching Cost - Block 7 | 252.25[20.50, 627.00] | 178.00[0.00, 575.00] |
| Planning - SWM - Strategy | 31[18, 47] | 30[11, 47] |
| *Memory* |  |  |
| Word memory - RAVLT - Immediate^ | 51.5[28, 71] | 52[33, 72] |
| Story memory - LM Immediate^ | 24[4, 40] | 27[10, 45] |
| Spatial working memory - SWM - Between errors | 17.0[0, 65] | 11.5[0, 65] |
| Visual learning - PAL - Total errors | 10.5[0, 114] | 7.5[0, 67] |
| *Verbal fluency - COWAT Corrected Score^* | 36[14, 83] | 38.5[21, 64] |
| ***Other ME/CFS Symptoms*** |  |  |
| General fatigue - MFI | 18[11, 20] | 18[11, 20] |
| Brainfog - MFTQ | 10.83[3.43, 19.00] | 8.00[3.00, 17.57] |
| Total symptoms - SSH | 31[6, 52] | 21[8, 44] |
| ***Microbiota*** |  |  |
| Streptococcus - Count | 7.76x10^6[3.16x10^5, 1.32x10^9] | 1.70x10^6(1, 3.98x10^8) |
| Bifidobacteria - Count | 7.94x10^2[1, 5.50x10^10] | 1(1, 1.38x10^9) |
| Lactobacillus - Count | 1[1, 1.38x10^9] | 1(1, 3.24x10^8) |
| Streptococcus - RA_aerobe | 63.62[5.93, 100.00] | 22.05[0.00, 100.00] |
| Streptococcus - RA_total | 0.00[0.00, 0.30] | 0.00[0.00, 0.28] |
| Bifidobacteria - RA_total | 0.00[0.00, 0.81] | 0.00[0.00, 031] |
| Lactobacillus - RA_total | 0.00[0.00, 0.46] | 0.00[0.00, 0.29] |
| ***Lactate*** |  |  |
| D:L Lactate Ratio | 0.25[0.02, 1.02] | 0.29[0.03, 0.92] |

| Lower scores reflect better symptoms for all clinical variables unless indicated by ^ |
| --- |
| Units of measurement for microbial variables: count = cfu/g back-transformed from Log10; RA_aerobe_ = percent distribution within total aerobic organisms determined via culture methods; RA_total_ = relative abundance of each genus within total bacteria determined via culture methods presented as a percentage. |

**Table S7.** Operational definitions of sleep terminology, measurement method and selected outcome variables

| **Sleep Terminology** | | **Abbrev.** | | **Unit** | **Definition** | | **Measurement method** | | | **Outcome variables** | | |
| --- | --- | --- | --- | --- | --- | --- | --- | --- | --- | --- | --- | --- |
|  | |  | |  |  | | **Actigraphy** | | **Sleep Diary** | **Selected Outcome Variables** | **Reason for selection** | **Reason for exclusion** |
| Main sleep interval | |  | |  | Period between *lights out* and *rise* | | *Actiware* use sleep/wake algorithms to determine mobility/ immobility and distinguish between *Rest* and *Sleep* intervals.  *Rest* interval: Estimate of period between *lights out* and *Rise*  *Sleep* interval: Differentiated by Actiware as an estimate of the period between sleep onset and FA | | N/A | N/A | | |
| Lights out | |  | | Time | Time participant turned off the lights to fall asleep | | Start of *Rest* interval | | Time turned off lights to fall asleep | N/A | | |
| Sleep onset | |  | | Time | Time participant fell asleep | | Start of *Sleep* interval (default detection algorithm = 10 immobile minutes) | | Actual time not used but could be approximated from participant’s estimate of SOL. | N/A | | |
| Final awakening | | FA | | Time |  | | End of *Sleep* interval (default detection algorithm = 10 immobile minutes) | | Final awakening time | N/A | | |
| Rise | |  | | Time | Time out of bed to start the day | | End of *Rest* interval | | Time out of bed to start the day | N/A | | |
| Epoch | |  | |  |  | | Selection of time to determine activity (immobile or mobile) counts  Default analysis properties were used for 30 second epoch lengths:  *Immobile* = < 2 activity counts  *Mobile* = > 2 activity counts  *Sleep* = activity counts < 40  *Wake* = activity counts > 40  *Invalid Time SW* = not enough data to determine sleep/wake scoring algorithm | |  | N/A | | |
| Duration of sleep episode | | DSE | | Minutes | Time between *lights out* and *rise* (FA in diary) | | *Rest* interval duration | | DSE = total time from *lights out* to *final awakening* calculated as the total of SOL + TST + WASO |  |  | Inability to accurately interpret directional change as beneficial or detrimental |
| Sleep efficiency | | SE | | % | The percentage of time spent sleeping during main sleep interval. | | SE = TST / (DSE – Invalid Time SW)) x 100 | | Ratio between total sleep time (TST) and duration of sleep episode (DSE), multiplied by 100  SE = (TST/ DSE) x 100 | - Actigraphy SE - Diary SE | Difference between ME and controls [42] |  |
| Sleep fragmentation index | | SFI | %+% | | Measure of restlessness, higher scores suggest increase sleep disturbance | | SFI = (Percent mobile + percent one minute immobile bouts) / number of immobile bouts during *Rest* interval.  Summary data from the Rest interval (rather than sleep) was chosen to reflect movement across the main sleep interval (i.e. *Rest*). | N/A | - Actigraphy SFI | Difference between ME and controls [42] |  |  |
| Sleep onset latency | | SOL | Minutes | | Time taken to fall asleep after initiating the intent to sleep (i.e. *lights out*) | | Time between start of *Rest* interval and start of *Sleep* interval determined by 10 immobile minutes. | Approximate minutes taken to fall asleep | - Actigraphy SOL - Diary SOL | Difference between ME and controls [42] |  |  |
| Time attempting to sleep after final awakening | | TASAFA | Minutes | | Interval between FA and *Rise* when attempting to fall back to sleep | | N/A | N/A  Information about intentions during this interval were not obtained. | N/A | | |  |
| Total sleep time | | TST | Minutes | | Amount of time asleep within main sleep interval | | Number of epochs scored as ‘sleep’ multiplied by length of epoch (30 seconds) within *Sleep* interval. | Time between *lights out* and FA minus SOL and WASO |  |  | Inability to accurately interpret directional change as beneficial or detrimental |  |
| Wake after sleep onset | | WASO | Minutes | | Length of time awake after falling asleep and before FA | | Number of epochs scored as ‘wake’ during *Sleep* interval, multiplied by epoch length (30 seconds) | Total time awake after sleep onset and before FA | - Actigraphy WASO - Diary WASO | Difference between ME and controls [42] |  |  |
| Wake bouts | | WB | Number | | Number of awakenings after falling asleep and before FA | | Number of continuous epoch blocks scored as ‘wake’ during *Sleep* interval | Total number of awakenings after sleep onset and before FA |  |  | Significant, positive correlations between Actigraphy WB and WASO and Diary WB and WASO, respectively. |  |

**Table S8.** Overview of cognitive test battery and selected outcome variables

|  | **Cognitive Skill** | **Test** | **Duration** | **Form** | **Alternate form** | **Outcome Variable** |
| --- | --- | --- | --- | --- | --- | --- |
| **Attention** | Sustained Visual Attention | Rapid Visual Information Processing (RVP) [43, 44] | 10 minutes | CANTAB  (touchscreen tablet) | N/A | - *RVP A’* ****Attention***   Measure of attention based on accuracy and sensitivity of participant detecting the number target sequence. Higher scores reflect better performance (scores from 0.00 to 1.00).   - *RVP Mean Latency* ****Processing speed***   Measure of processing speed when accurately detecting the target sequence. Lower scores suggest faster speed. |
| **Executive Functioning** | Visual attention, reaction time and inhibition | Attention Switching Task (AST) [44] | 8 minutes | CANTAB  (touchscreen tablet) | N/A | - *AST Median Switching Cost – Block 7* ****Cognitive flexibility***   Measure of cognitive flexibility based on the difference in reaction times across congruent and incongruent trials. Higher scores indicate slower reaction time on switching (incongruent) compared to non-switching (congruent) trials. A score of zero indicates the same reaction speed on both types of trials. Block 7 was selected to reduce practice effects. |
|  | Visual working memory and attention | Spatial Working Memory (SWM) [44–46] | 8 minutes | CANTAB  (touchscreen tablet) | N/A | - *SWM – Between Errors* ****Spatial working memory***   Measure of visual memory and attention based on the total number of errors a respondent makes by incorrectly checking a box where a token has previously been found during assessed trials. Lower scores indicate better performance.   - *SWM – Strategy Score* ****Planning***   Measure of planning based on trials with more than 6 boxes. Lower scores indicate better strategy. |
| **Visual Memory** | Visual learning and memory | Paired Associate Learning (PAL) [44] | 8 minutes | CANTAB  (touchscreen tablet) | Available | - *PAL – Total Errors (adjusted)* ****Visual learning***   Measure of visual memory and learning based on the number of errors on both completed and incompleted (if test aborted due to failed attempts). Lower scores indicate better memory. |
| **Verbal Memory** | Verbal Memory (list learning) | Rey Auditory Verbal Learning Test (RAVLT) Form A [47–49] | 8 minutes (with 30 minute delay) | Pen and paper | Form B developed by Jones-Gotman, Sziklas & Majdan (1993, in [49]) | - *RAVLT Total Score Trials 1-5* (raw) ****Word memory***   Measure of word memory for unrelated auditory information. Higher scores indicate better performance. More reliable and robust outcome measure rather than reliance on individual trial scores [30, 50]. |
|  | Verbal Memory (Story) | Form A: Weschler Memory Scales (WMS-IV): Logical Memory Subscale [51] | 8 minutes (with 30 minute delay) | Pen and paper | Form B: Morris Revision-Fourth Edition (MR-IV; [52]) | - *Story Memory Total Immediate* (raw) ****Story memory***   Measure of story/lexical memory for conceptually related information. Total raw scores were used to enable comparison between forms. Higher scores indicate better performance. Selected because lower practice effects than delayed total score. |
| **Verbal fluency** | Verbal Fluency | Controlled Oral Word Association Test (COWAT) [53, 54]Form A: C, F, L | 3 x 1 minute trials | Pen and paper | Form B: P, R, W | - *COWAT Corrected Score* ****Verbal fluency***   Measure of verbal fluency and word retrieval. Higher scores indicate better performance. |

** Denotes outcome variable label used within main body of the article.*

**Table S9.** Scoring procedures for managing missing and ambiguous data.

|  | | **Missing Data** | **Ambiguous Data** |
| --- | --- | --- | --- |
| **Sleep Diary** | |  |  |
| Duration of sleep episode (final awakening) | DSE | Not calculated if missing lights out and FA times |  |
| Sleep efficiency | SE | Not calculated if missing TST or DSE |  |
| Sleep onset latency | SOL | Not calculated if missing | - Mean calculated when a range was given (e.g. 40-60 minutes = 50 minutes). |
| Total sleep time | TST | Not calculated if missing lights out, FA, SOL or WASO |  |
| Wake after sleep onset | WASO | Not calculated if the number of estimated wake lengths (in minutes) was not equivalent to the number of wake bouts for that night. | - Mean calculated when a range was given. - “a few” = 3 minutes |
| Wake bouts | WB | Not calculated if missing | - Mean calculated when a range was given. |
| Sleep Quality |  | Not calculated if missing |  |
| Rested |  | Not calculated if missing |  |
| *Weekly Mean scores for all above sleep diary variables* |  | Only calculated when data was available for **4 of the 7 nights** |  |
|  |  |  |  |
| **Questionnaires** |  |  |  |
| MAC_Positive |  | Not calculated if missing any items | The middle value was calculated if two responses were provided to the one item and all other items were completed.  If two responses were provided and the next item left blank (i.e. possible error of placement), both items were calculated as missing data. |
| MAC_Negative |  | Not calculated if missing any items | As above for MAC_Positive |
| MFTQ_Brainfog |  | Not calculated if missing any items | As above for MAC_Positive |
| *Weekly Mean scores for all above day diary variables* |  | Only calculated when data was available for **4 of the 7 nights** |  |
|  |  |  |  |
| SSH_Total Symptoms |  | Not calculated if missing any items |  |
|  |  |  |  |
| DASS-21 |  | Individual subscales were not calculated if missing any items. |  |
|  |  |  |  |
| MFI-20 |  | Individual subscales were not calculated if missing any items.  Total score not calculated if missing one or more subscale scores. |  |
|  |  |  |  |
| POMS |  | Individual subscales were not calculated if missing any items.  Total score not calculated if missing one or more subscale scores. | An average of all other FI items was used for the item ‘bushed’ when a participant indicated they were unsure of the meaning (i.e., ‘?’) but answered all other items on the POMS. |
|  |  |  |  |
| PSQI |  | PSQI factors not calculated if any missing data.  PSQI_Total not calculated if missing any of the PSQI factors. | Mean calculated when a range was given for items 1-4. |
|  |  |  |  |
| ISI |  | ISI_Total not calculated if missing any items. |  |
| **Faecal microbial analysis** |  | An arbitrary value (1) was used for Count variables to indicate that the analysed genera was not detected through culture methods but was not missing. This was also required for Log10 transformations.  Inadequate (due to collection error) or unreturned samples were entered as missing. |  |
| **Urine lactate analysis** |  | Only cases with both baseline and post-intervention urine samples were analysed.  Inadequate (due to collection error) or unreturned samples were entered as missing. |  |
